# An endothelial-centered regulatory framework reveals context-dependent roles of MYLK in lung adenocarcinoma

**DOI:** 10.3389/fimmu.2026.1719296

**Published:** 2026-04-01

**Authors:** Rui Li, Xiao Liu, Chen Huo, Jian-Ping Li, Yu-Xin Zhang, Shuang-Teng Liu, Yi-Qing Qu

**Affiliations:** Department of Pulmonary and Critical Care Medicine, Qilu Hospital of Shandong University, Jinan, China

**Keywords:** endothelial cells, lung adenocarcinoma, MYLK, single-cell RNA sequencing, trans-endothelial-migration

## Abstract

**Background:**

Lung adenocarcinoma (LUAD) is shaped by the tumor microenvironment, yet endothelial cell (EC) regulatory programs and their biological roles remain insufficiently defined.

**Methods:**

We analyzed scRNA-seq data to map EC-associated programs and applied hdWGCNA to identify EC modules and communication patterns. Network pharmacology integrated EC-module genes with LUAD-related targets to prioritize MYLK. MYLK expression and function were evaluated by RT-qPCR, immunohistochemistry, and gain-/loss-of-function assays in endothelial and LUAD cell models. We then performed network-based in silico knockout in LUAD tumors (GSE164789) and exploratory immune-cell eQTL analysis.

**Results:**

EC modules were enriched for junction organization, angiogenesis, and immune-related pathways, with extensive epithelial-stromal-endothelial interactions. Network pharmacology nominated MYLK as an EC-linked LUAD candidate. MYLK expression was reduced in LUAD and associated with unfavorable clinical outcomes. In endothelial cells, MYLK perturbation altered junction integrity and trans-endothelial tumor cell migration; in LUAD cells, MYLK gain/loss affected migration, invasion, and proliferation. In silico knockout of MYLK produced regulatory shifts enriched for tight junction organization, endothelial apoptosis, angiogenesis, vascular permeability, and vascular/cancer-related pathways. Immune-cell eQTL analysis identified an association between increased MYLK expression in dendritic cells and elevated LUAD risk.

**Conclusions:**

These findings define an endothelial-centered regulatory framework in LUAD and highlight the context-dependent, cell-type–specific roles of MYLK at the tumor–endothelial–immune interface.

## Introduction

Lung adenocarcinoma (LUAD) constitutes a prominent subtype of non-small cell lung cancer (NSCLC) and is distinguished by its aggressive characteristics and unfavorable prognosis (1). This condition imposes a significant burden on patients and healthcare infrastructures worldwide, resulting in elevated morbidity and mortality rates (2). The existing treatment options, which include surgical intervention, chemotherapy, and targeted therapies, demonstrate only modest effectiveness, particularly during the later stages of the disease (3–7). This situation underscores the critical need for innovative therapeutic approaches and diagnostic methodologies. Notwithstanding progress in genomic profiling and targeted therapies, a considerable knowledge gap persists regarding the intricate tumor microenvironment and the cellular interactions that facilitate LUAD progression (8–10). Some investigations seek to bridge these gaps by utilizing single-cell RNA sequencing alongside sophisticated bioinformatics analyses to unravel the cellular diversity and communication networks present within the LUAD microenvironment (11–13). Despite advances in surgical resection, targeted therapy, and immunotherapy, the prognosis of patients with advanced LUAD remains unsatisfactory. Increasing evidence indicates that LUAD progression is not solely driven by malignant epithelial cells, but is critically shaped by the tumor microenvironment, which orchestrates tumor growth, dissemination, and immune evasion.

By utilizing the advanced techniques of single-cell RNA sequencing, scientists are exploring the multifaceted gene expression profiles, functional characteristics, and complex cellular interactions present among various subpopulations in LUAD (14–18). Endothelial cells (ECs) are crucial in the advancement of tumors. At present, the vascular endothelial growth factor (VEGF) pathway is the primary focus of anti-angiogenic therapeutic strategies (19, 20). Research has demonstrated that the development of new blood vessels within the tumor microenvironment represents a vital milestone in the progression of solid tumors, serving as a prerequisite for tumor invasion and metastasis (21). A notable aspect of the angiogenesis associated with tumors is the induction of pro-angiogenic factors in endothelial cells (22), which become activated and give rise to diverse subpopulations. Single-cell RNA sequencing (scRNA-seq) has enabled high-resolution dissection of cellular heterogeneity and intercellular communication in solid tumors. When combined with network-based approaches such as high-dimensional weighted gene co-expression network analysis (hdWGCNA), scRNA-seq data allow the identification of cell-type–specific transcriptional modules and regulatory hubs that may be overlooked in bulk analyses (23–25). However, systematic efforts to define EC-centered regulatory networks and to functionally validate their key drivers in LUAD remain limited.

In this study, we integrated single-cell and network-based analyses to identify endothelial-associated gene modules and communication patterns in LUAD. Through network pharmacology analysis, myosin light chain kinase (MYLK) was prioritized as an EC-associated candidate linking endothelial regulation with tumor biology. We further observed that MYLK expression was reduced in LUAD and associated with unfavorable clinical outcomes, motivating functional investigation across endothelial, tumor, and immune cellular contexts. MYLK expression and function were subsequently examined using gain- and loss-of-function approaches in endothelial and LUAD cell models, focusing on endothelial junction integrity and tumor cell behavior. To complement these findings, we performed network-based in silico knockout analysis to assess tumor-level transcriptional perturbations associated with MYLK disruption, together with exploratory single-cell eQTL analyses to evaluate immune cell–specific associations. This stepwise framework was designed to define the context-dependent roles of MYLK at the tumor–endothelial–immune interface in LUAD, rather than to establish therapeutic efficacy.

## Materials and methods

### Data collection

We downloaded the single cell RNA sequencing (scRNA-seq) data GSE149655 from GEO (Gene expression omnibus) database. GSE140797, GSE117049, GSE115002 datasets in LUAD were also downloaded. We collected 10 paired LUAD tissues and adjacent normal tissues from Qilu hospital of Shandong university between January 2023 and August 2024. None of the patients underwent chemotherapy or radiotherapy prior to their surgical procedures. All tissues were stored at 4 °C using RNA wait (Top0866, Biotopped, Beijing, China) reagent for future use. The study received ethical approval from the Research Ethics Committee of Qilu Hospital of Shandong University, and all participants provided written informed consent following the declaration of Helsinki guidelines. Ethical approval was obtained from the Research Ethics Committee of Qilu Hospital of Shandong University (approval number: KYLL-2021(KS)-521). All samples were anonymized prior to analysis.

### Single-cell RNA-seq preprocessing, quality control, clustering, and annotation

Single-cell RNA-seq data were processed in R (v4.4.3) using Seurat (v5.2.1). Raw 10× Genomics count matrices were imported with Read10X, and a Seurat object was constructed using CreateSeuratObject with min.cells = 3 and min.features = 100. The percentage of mitochondrial transcripts was calculated using PercentageFeatureSet with the pattern “^MT-”. Cells were retained if they met the quality-control criteria of nFeature_RNA > 50 and percent.mt < 15%. After quality-control filtering (nFeature_RNA > 50 and percent.mt < 15%). Following quality-control filtering, the numbers of retained cells were summarized across samples and reported for transparency (**Supplementary Tables S1A, E**). Data were normalized using LogNormalize (scale factor 10,000). Highly variable genes were identified using the vst method, selecting 1,500 features. The data were scaled (ScaleData) and subjected to principal component analysis (RunPCA) with 20 principal components. Potential batch effects attributable to sample-of-origin (orig.ident) were assessed and corrected using Harmony (v1.2.3). Principal components (dims 1-20) were selected based on JackStraw resampling. Graph-based clustering was performed using FindNeighbors (dims 1-20) followed by FindClusters (resolution 0.6; candidate resolutions 0.5–1.2 were explored). After graph-based clustering, the number of cells assigned to each cluster was recorded to document cluster size distribution (**Supplementary Table S1D**). Cells were visualized by t-SNE (RunTSNE, dims 1-20). Cluster marker genes were identified using FindAllMarkers with min.pct = 0.25, logfc.threshold = 1, and only.pos = FALSE. Significant markers were defined as those with |avg_log2FC| > 1 and adjusted *P* < 0.05. Cells were grouped by tissue status with Control = normal samples and Treat = tumor samples. Cell type annotation was performed using SingleR (v2.8.0) with reference datasets provided by celldex (v1.16.0) and additional immune/hematopoietic references. Cluster-level labels were assigned by comparing cluster expression profiles across multiple reference atlases and then propagated to single cells. Cell numbers for each annotated cell type and their distribution across tissue conditions were summarized for descriptive purposes (**Supplementary Tables S1B, C**). Within each annotated cell type, differential expression between tumor (Treat) and normal (Control) cells was performed using FindMarkers (logfc.threshold 0.1) when both groups contained >10 cells, and significant genes were defined using |avg_log2FC| > 1 with adjusted *P* < 0.05.

### Cell-cell communication analysis

Cell-cell communication analysis was performed using CellChat (v2.1.2) in R (v4.4.3). Normalized scRNA-seq expression data and Seurat-derived cell type annotations were integrated to create a CellChat object. Human ligand–receptor interactions were retrieved from CellChatDB.human, and putative interactions were inferred using the standard CellChat workflow. Communications involving cell groups with fewer than 10 cells were excluded (min.cells = 10). Communication probabilities were computed and summarized at the ligand-receptor level, and results were visualized using bubble plots and network diagrams.

### High-dimensional WGCNA in endothelial cells

High-dimensional weighted gene co-expression network analysis was conducted in endothelial cells (ECs) using hdWGCNA (v0.4.5) and WGCNA (v1.73) in R (v4.4.3). To improve co-expression estimation robustness for sparse single-cell data, metacells were constructed prior to network inference. Analyses were performed with a fixed random seed (set.seed(12345)), and multithreading was enabled using enableWGCNAThreads (nThreads = 5). Genes were selected using the fraction-based procedure implemented in SetupForWGCNA (gene_select = “fraction”), retaining genes expressed in at least 5% of cells (fraction = 0.05). Metacells were generated using MetacellsByGroups by grouping cells according to cell type and sample group (Type; normal vs tumor), with KNN computed on the Harmony reduction (reduction = “harmony”). Harmony was used to assess potential batch effects; clustering was performed on the PCA embedding (dims 1–20). Metacell construction used k = 25 nearest neighbors and allowed a maximum of 10 shared cells between metacells (max_shared=10). Metacell expression matrices were normalized using NormalizeMetacells. The expression matrix for network construction was then set for endothelial cells (group_name = “Endothelial cells”, group.by = “cell_type”, assay RNA, layer data) using SetDatExpr. Soft-thresholding power was evaluated using TestSoftPowers with a signed network (networkType = “signed”). A co-expression network was constructed using ConstructNetwork with soft power = 8 (topological overlap matrix saved per cell type). Module eigengenes were computed using ModuleEigengenes with Type as the grouping variable (group.by.vars = “Type”), and eigengene-based connectivity (kME) was calculated using ModuleConnectivity. Hub genes were identified using GetHubGenes (top 25 hub genes per module). Module activity scores were computed using ModuleExprScore (n_genes = 25, Seurat method) and visualized on low-dimensional embeddings. Inter-module correlations and module–cell type relationships were summarized using correlograms, dot plots, and violin plots. All hdWGCNA parameters are provided above to facilitate exact reproduction.

### Functional and pathway enrichment analysis of endothelial cells-related core module genes

To explore functional associations of EC-related module genes, we performed the GO and KEGG (Kyoto Encyclopedia of Genes and Genomes) analysis. We used the R packages including clusterProfiler, org.Hs.eg.db, enrichplot, ggplot2, circlize, RColorBrewer, dplyr, ComplexHeatmap to conduct the GO and KEGG analysis.

### Differential expression analysis of endothelial module genes in bulk cohorts

Bulk datasets GSE140797, GSE117049, and GSE115002 were merged and analyzed in R using limma, dplyr, pheatmap, and ggplot2. To mitigate batch effects across cohorts, batch correction was performed using the sva package (ComBat), with study identity used as the batch variable. Endothelial module genes were extracted from the merged expression matrices, and differential expression analyses were conducted between LUAD and normal samples using limma. Results were visualized using heatmaps and box plots.

### ROC analysis of differentially expressed ECs key genes

Receiver operating characteristic (ROC) analysis was performed to quantify the gene-level ability of EC-related genes to distinguish LUAD from normal samples. ROC curves were generated separately for each gene, such that each curve reflects the discriminative behavior of a single gene rather than a multi-gene diagnostic classifier. Gene-level ROC/AUC analyses were first conducted in the merged discovery cohort. For external validation, the same ROC-based procedure was applied to an independent bulk RNA-seq dataset (GSE85841). A fixed set of 23 EC-related genes was defined based on the discovery cohort and carried unchanged into the validation cohort. No gene re-selection, refitting, re-ranking, or additional parameter tuning was performed during validation. ROC/AUC values were computed using the same analytical procedure in both cohorts. This analysis was designed to assess gene-level discrimination in an exploratory manner and was not intended to evaluate diagnostic classifiers or clinical predictive performance.

### Exploratory machine learning analysis for model comparison and feature prioritization

Expression data and sample group information were used for exploratory machine learning (ML) analyses. Data were randomly split into 70% training and 30% testing sets with a fixed random seed (123). Ten ML algorithms were evaluated, including PLS, RF, DTS, SVM, logistic regression, KNN, XGBoost, GBM, NeuralNet, and glmBoost. Given the merged nature of public datasets, potential batch effects, and prior feature selection, these ML analyses were conducted solely to compare relative model behavior and to support feature prioritization, rather than to develop or evaluate diagnostic classifiers. Model performance was summarized using ROC curves and AUC values in the held-out test set, with AUC interpreted only as an apparent, model-specific metric under this exploratory setup. The model with the highest apparent AUC (SVM) was used as a ranking tool to extract feature importance and prioritize EC-associated genes for downstream analysis. Feature importance was not interpreted as evidence of predictive power, causality, or generalizable diagnostic performance. No nested cross-validation, group-wise splitting, or fold-internal feature selection was implemented, and performance metrics derived from this analysis are not intended to estimate real-world classification accuracy.

### Immune infiltration and correlation analysis

Immune cell infiltration was estimated from the merged bulk expression data (GSE117049, GSE140797, GSE115002) using CIBERSORT with the default signature matrix (LM22) and standard settings. Genes prioritized by feature importance from the best-performing ML model were correlated with inferred immune cell fractions using Spearman correlation, and results were visualized using linkET plots.

### SsGSEA

Hallmark gene sets were used for ssGSEA scoring in merged bulk datasets. Differences in ssGSEA scores between normal and LUAD groups were visualized using box plots. Correlations between prioritized genes and ssGSEA pathway scores in LUAD samples were calculated and displayed as heatmaps.

### In silico identification of MYLK-associated compounds and molecular docking analysis

LUAD-related genes were compiled by integrating data from GeneCards, CTD, TTD, and OMIM. Candidate compounds were retrieved from Herb 2.0 and screened by SwissADME using standard drug-likeness criteria (molecular weight < 500, H-bond donors < 5, H-bond acceptors < 10, rotatable bonds < 10, LogP < 5, and high gastrointestinal absorption; BBB permeability was also considered). Docking between MYLK and a candidate compound (Coumingidine) was performed using CB-Dock2 with protein structures obtained from RCSB PDB and ligand structures from PubChem. Among the compounds screened, Coumingidine was identified as a putative MYLK-compatible compound based on in silico docking and dynamics simulations. However, Coumingidine could not be synthesized due to the unavailability of its natural source material (from African trees) (26), and the long synthesis cycle made it impractical for further experimental testing. Therefore, ML-7, a well-known MYLK inhibitor, was selected to further investigate MYLK’s role in endothelial cell function. Docking and molecular dynamics analyses were conducted solely to explore potential physical compatibility between MYLK and Coumingidine and were not intended to demonstrate biochemical target engagement.

### Molecular dynamics simulations

Molecular dynamics (MD) simulations of the Coumingidine–MYLK complex were performed using GROMACS 2022.3. Ligand parameters were generated using AmberTools 22 with GAFF. RESP charges were computed using Gaussian 16W and incorporated into topology files. Simulations were conducted at 300 K and 1 bar for 100 ns using the Amber99sb-ildn force field and TIP3P water model. Sodium ions were added to neutralize the system. Energy minimization was performed using steepest descent, followed by equilibration prior to production runs. MD simulations were used to assess pose stability under idealized conditions and do not substitute for biochemical or cellular target engagement assays.

### Validation of MYLK expression in LUAD

MYLK expression was evaluated in ECs within GSE149655 using scatter, bubble, and violin plots. MYLK expression was further analyzed using GEPIA and validated in 10 paired LUAD and adjacent normal tissues. RNA was isolated from Beas-2B and LUAD cell lines (A549, H1975, H1299) using Fastgen reagents, reverse transcribed using the EvoM-MLV RT kit, and quantified by RT-qPCR using SYBR Green with GAPDH as reference. Relative expression was calculated using the 2^−ΔΔCt method. Primer sequences are provided in **Table 1**. All human cell lines were authenticated using STR (or SNP) profiling within the last three years. Correlations between MYLK and PECAM1 (CD31) were assessed in GEPIA.

**Table 1:** The primer sequences of GAPDH and MYLK.

Primer	sequences (5’->3’)
Homo-GAPDH-Forward	GTGGACCTGACCTGCCGTCTAG
Homo-GAPDH-Reverse	GAGTGGGTGTCGCTGTTGAAGTC
Homo-MYLK-Forward	GTTGGTGCTGATGGTGGTGGTAG
Homo-MYLK-Reverse	GTCCTCGCCGTCTTCCTCCTC

### Immunohistochemistry

Formalin-fixed, paraffin-embedded paired LUAD specimens, including tumor tissues and matched adjacent non-tumor tissues, were subjected to IHC analysis. Tissue sections were deparaffinized and rehydrated through a graded ethanol series (100%, 95%, 80%, 75%) and rinsed three times with phosphate-buffered saline (PBS). Heat-induced antigen retrieval was performed in EDTA buffer (pH 9.0) using microwave treatment. Endogenous peroxidase activity was quenched with hydrogen peroxide, and nonspecific binding sites were subsequently blocked with normal serum. The sections were incubated overnight at 4 °C with a primary antibody against MYLK (CY5428, ABWAYS) at a dilution of 1:200. After PBS washes, HRP-conjugated secondary antibody was applied, and immunoreactivity was visualized using a DAB chromogenic substrate. Slides were counterstained with hematoxylin, briefly differentiated in 1% hydrochloric acid ethanol for 2 s, rinsed thoroughly, dehydrated through an ascending ethanol gradient (75%, 80%, 95%, 100%), cleared using a commercial xylene substitute, mounted with coverslips, and examined under a Nexcope NIB-610 inverted microscope.

### Endothelial functional validation (junction integrity and trans-endothelial migration)

Endothelial junction integrity and trans-endothelial migration were assessed following genetic manipulation or pharmacologic inhibition of MYLK in HUVECs. Human umbilical vein endothelial cells (HUVECs) were maintained at 37 °C in a humidified incubator with 5% CO_2_ under standard culture conditions. For MYLK gain-of-function, HUVECs were transfected with a MYLK overexpression plasmid (OE-MYLK: MYLK [NM_053025.4]-FLAG-C in pcDNA3.1) or the corresponding negative control plasmid (NC-MYLK: pcDNA3.1). For MYLK loss-of-function, HUVECs were transfected with two independent MYLK-targeting siRNAs (si-1130 and si-5407) or a non-targeting control siRNA (si-NC). Unless otherwise stated, downstream assays were initiated 24 h post-transfection. For junctional immunofluorescence, transfected HUVECs were seeded on sterile glass coverslips in 24-well plates at 6×10^4^cells/well and cultured until a confluent monolayer formed (48 hours). Cells were washed with PBS, fixed with 4% paraformaldehyde for 15 min at room temperature, permeabilized with 0.2% Triton X-100 for 10 min, and blocked with 1% BSA in PBS for 1 hour. Coverslips were incubated overnight at 4 °C with primary antibodies against VE-cadherin (mouse; Proteintech; 1:500) and ZO-1 (rabbit; ABclonal; 1:500). After PBS washes, samples were incubated for 1 h at room temperature in the dark with secondary antibodies (1:500): goat anti-mouse Alexa Fluor 594 and goat anti-rabbit Alexa Fluor 488. Nuclei were counterstained with Hoechst 33342 (1 μg/mL, 10 min). Coverslips were mounted using an anti-fade mounting medium and stored at 4 °C in the dark prior to imaging. Trans-endothelial migration assays were conducted using Transwell inserts (24-well format) with 8-μm pore-size membranes under two conditions: genetic manipulation alone or combined with pharmacologic inhibition of MYLK. Transfected HUVECs (NC-MYLK or OE-MYLK) were seeded onto the inserts at a density of 6 × 10^4^cells per insert in DMEM/F12 supplemented with 10% FBS, with identical medium added to both the upper and lower chambers. After 24 h, when a confluent endothelial monolayer had formed, transmigration assays were initiated. For assays without pharmacologic pretreatment, the medium in the upper chamber was removed and untreated H1299 cells (3×10^4^ cells per insert), resuspended in serum-free RPMI-1640, were added to the upper chamber. The lower chamber medium was simultaneously replaced with RPMI-1640 containing 20% FBS (600μL per well) to establish a chemotactic gradient. For pharmacologic inhibition experiments, the upper chamber medium was replaced with fresh DMEM/F12 containing either ML-7 (1 μM) or vehicle (volume-matched DMSO), generating four experimental groups: NC-MYLK + DMSO, NC-MYLK + ML-7, OE-MYLK + DMSO, and OE-MYLK + ML-7. ML-7 (Selleck Chemicals) was prepared as a 10 mg/mL stock solution in DMSO and diluted to a final working concentration of 1 μM. HUVEC monolayers were pretreated for 2 h at 37 °C, after which the ML-7- or DMSO-containing medium was removed. Untreated H1299 cells (3×10^4^ cells per insert) in serum-free RPMI-1640 were then added to the upper chamber, and the lower chamber medium was replaced with RPMI-1640 containing 20% FBS (600 μL per well). After 20 hours of incubation, non-migrated cells on the upper surface of the membrane were removed. Cells that had migrated to the underside of the membrane were fixed and stained with crystal violet. Migrated cells were quantified by counting at least five randomly selected microscopic fields per insert, and values were averaged across three independent experiments. These *in vitro* assays were designed to assess endothelial barrier-related phenotypes and were not intended to model *in vivo* angiogenesis or immune regulation.

### Tumor cell-intrinsic functional assays of MYLK

For MYLK overexpression, H1299 cells were transfected with either a MYLK overexpression construct (MYLK-OE: MYLK(NM_053025.4)-FLAG-C in pcDNA3.1) or a control vector (NC-MYLK: pcDNA3.1). For MYLK knockdown, H1975 cells were transfected with two independent siRNAs targeting MYLK (si-1130-MYLK and si-5407-MYLK) or a control siRNA (si-NC). Transfection was performed using the JET Transfection Reagent according to the manufacturer’s instructions. siRNA sequences are provided in **Table 2**. Cell migration and invasion assays were performed using Transwell chambers with or without Matrigel coating. H1299 cells (NC-MYLK and MYLK-OE) and H1975 cells (si-NC, si-1130-MYLK, si-5407-MYLK) were seeded into the upper chamber. After 24 h, cells that migrated/invaded to the lower membrane surface were fixed, stained, and counted. Cell proliferation was assessed using an EdU incorporation assay (BeyoClick EdU-C0078S). H1299 cells (NC-MYLK and MYLK-OE) and H1975 cells (si-NC, si-1130-MYLK, si-5407-MYLK) were incubated with EdU for 24 h. Cells were processed according to the manufacturer’s instructions, and the percentage of EdU-positive cells was quantified using a Nikon Eclipse Ti2 microscope.

**Table 2:** The sequences of siRNAs of MYLK.

Name	Nucleotide sequence (5’-3’)	
si-1130-MYLK	sense	GAGGUGACCAAUGUAAUCUTT
	Antisense	AGAUUACAUUGGUCACCUCTT
si-5407-MYLK	sense	GCUGAAGAAAGAUAUGAAATT
	Antisense	UUUCAUAUCUUUCUUCAGCTT

### Microscopy

Brightfield images for IHC were acquired using a Nexcope NIB-610 inverted microscope with a 10× objective lens (scale bar: 200 μm). Brightfield images for tumor cell migration, invasion, and trans-endothelial migration assays were obtained using the same microscope with a 20× objective lens (scale bar: 100 μm). Immunofluorescence images for endothelial junction staining were captured using a Nikon upright fluorescence microscope with a 40× objective lens (scale bar: 20 μm). EdU proliferation images were acquired using a Nikon Eclipse Ti2 microscope with a 20× objective lens (scale bar: 100 μm). All scale bars were calibrated using Image J software based on objective magnification and image resolution and are positioned in the lower-right corner of each image panel.

### Network-based in silico knockout of MYLK in tumor samples

To investigate transcriptional programs associated with MYLK perturbation at the tumor level, we performed a network-based in silico knockout analysis using the scTenifoldKnk framework. scTenifoldKnk infers single-cell gene regulatory networks and computationally removes the regulatory influence of a target gene, thereby simulating a virtual gene knockout at the network level rather than transcript-level knockdown. Four samples (GSM5019481, GSM5019482, GSM5019483, and GSM5019484) from the GSE164789 dataset were analyzed independently. For tumor sample, MYLK was designated as the knockout gene, and scTenifoldKnk was applied to generate perturbed gene regulatory networks. Differential network-derived gene scores were then calculated by comparing the MYLK virtual knockout condition with the corresponding original network state. Genes showing significant changes in network influence were identified and subjected to downstream Gene Ontology (GO) and Kyoto Encyclopedia of Genes and Genomes (KEGG) enrichment analyses using the clusterProfiler package. This analysis was designed to evaluate pathway-level convergence associated with MYLK network perturbation rather than to infer direct genetic knockdown effects or cell-type-specific causality.

### Single-cell eQTL analysis

Single-cell eQTL (sceQTL) data for 14 immune cell types were obtained from the ONEK1K resource. MYLK-associated sceQTL signals were extracted and analyzed using LUAD GWAS summary statistics (GWAS Catalog: GCST90568463), focusing on MYLK signals in two immune cell types. These analyses were not designed for causal inference and should be interpreted as hypothesis-generating associations.

### Statistical analysis

All experiments were independently repeated three times. Data are presented as mean ± SEM unless otherwise stated. Statistical analyses were performed using GraphPad Prism 9.0. Two-group comparisons used two-tailed Student’s t-test, and comparisons among three or more groups used one-way ANOVA with appropriate *post hoc* multiple-comparison testing when indicated. Bioinformatics analyses were performed in R (v4.4.3). A *P* value < 0.05 was considered statistically significant. All computational analyses were performed in R (v4.4.3) using Seurat (v5.2.1), Harmony (v1.2.3), SingleR (v2.8.0), celldex (v1.16.0), CellChat (v2.1.2), hdWGCNA (v0.4.5), and WGCNA (v1.73).

## Results

### Single-cell landscape and cell–cell communication in LUAD

**Figure 1:**
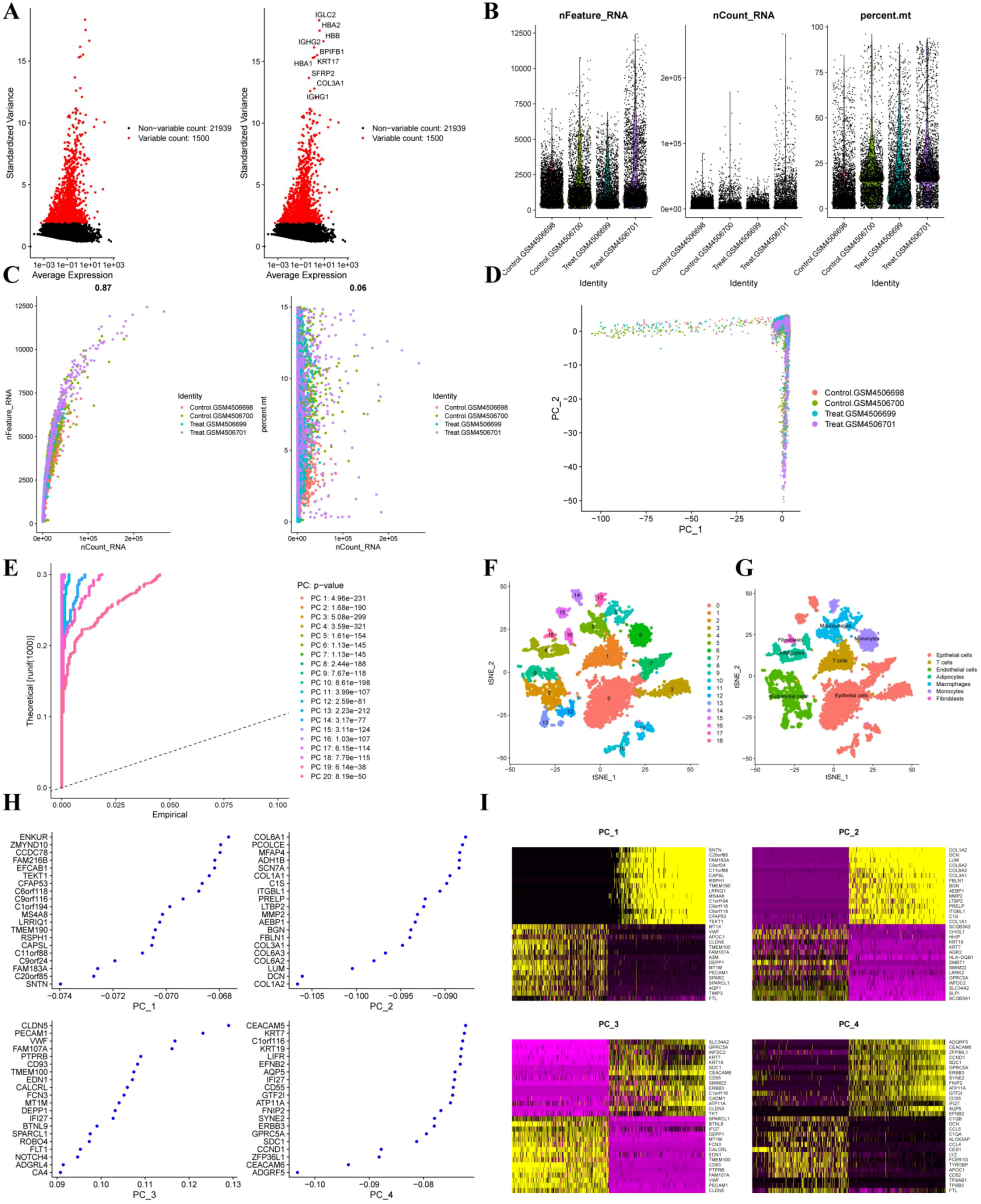
Single-cell RNA sequencing analysis of GSE149655. **(A)** Feature variance plot showing highly variable genes across individual cells in the GSE149655 dataset. Red dots indicate genes with high coefficients of variation, whereas black dots represent genes with lower variability. **(B)** Quality-control metrics, including detected gene counts, sequencing depth, and mitochondrial gene content, across the four samples (GSM4506698, GSM4506700, GSM4506699, GSM4506701). **(C)** Correlation analysis between sequencing depth, detected gene number, and mitochondrial gene content across samples. **(D)** Principal component analysis (PCA) of all cells from the four samples. **(E)** Statistical significance of principal components (PCs) determined by JackStraw analysis. **(F, G)** Unsupervised clustering and cell type annotation based on the first 20 principal components. **(H)** Scatter plot displaying the top feature genes contributing to the major principal components. **(I)** Heatmap showing the top feature genes associated with the major principal components.

**Figure 2:**
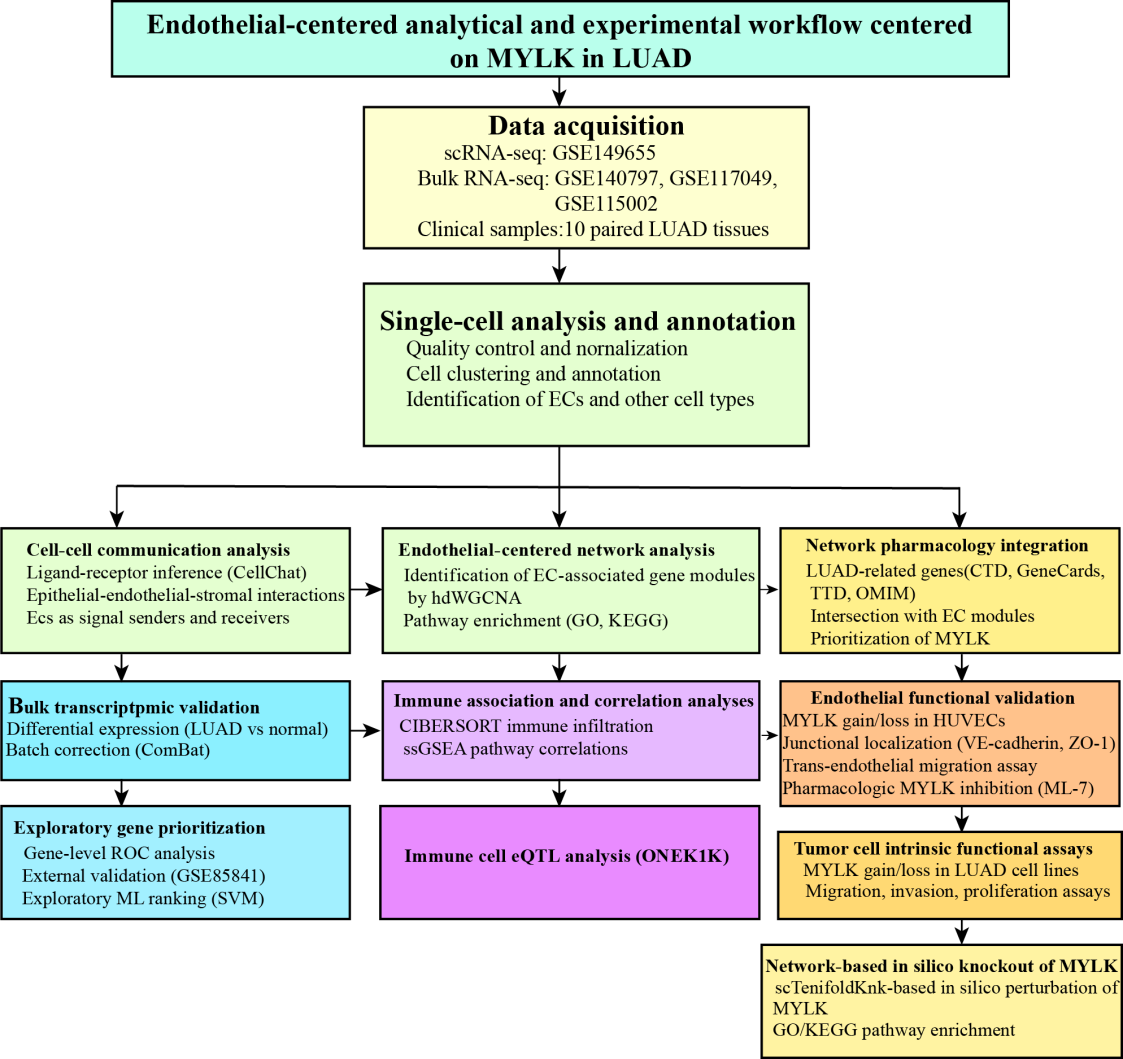
Overview of the analytical workflow of the study. Schematic diagram illustrating the integrated single-cell, network-based, and experimental framework used in this study, including single-cell RNA sequencing analysis, cell–cell communication inference, hdWGCNA module identification, bulk transcriptomic validation, exploratory machine learning–based gene prioritization, network pharmacology analysis, and functional validation of MYLK in endothelial and tumor cell models.

**Figure 3:**
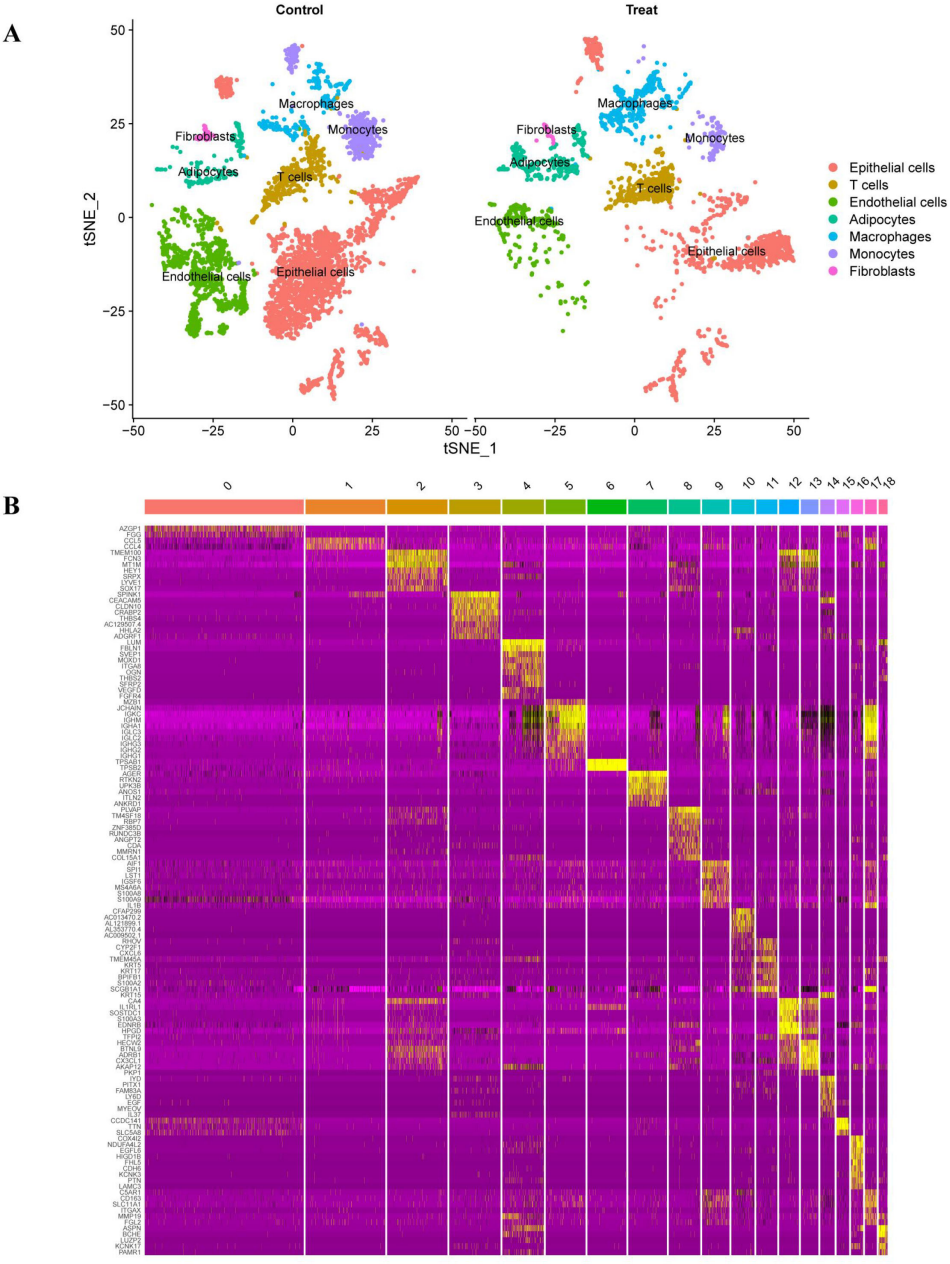
Single-cell annotation and cluster-level differential expression. **(A)** Distribution of annotated cell types in normal and LUAD samples. **(B)** Heatmap illustrating the most significantly differentially expressed genes across the 18 identified cell clusters.

**Figure 4:**
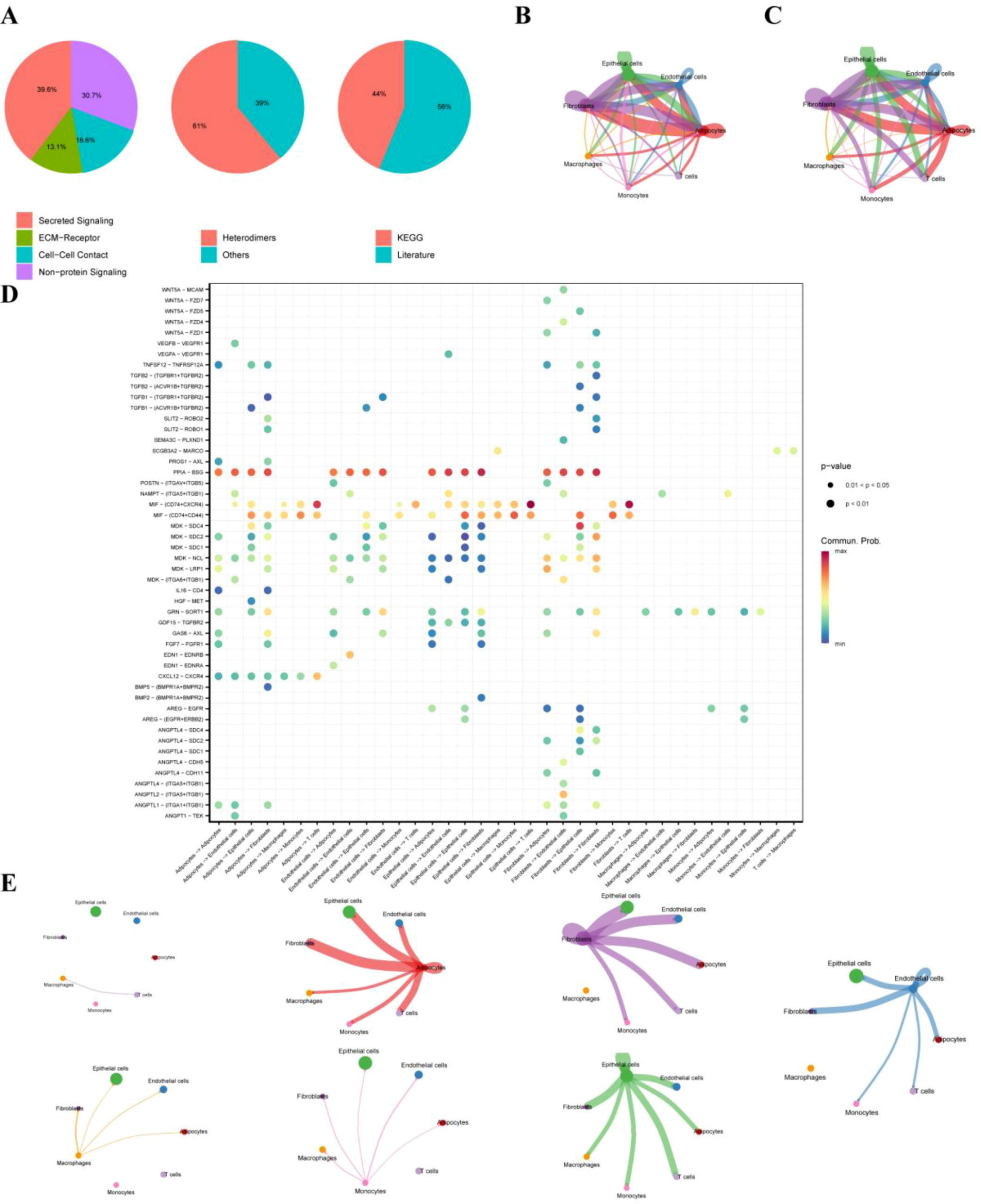
Cell-cell communication analysis in LUAD. **(A)** Classification of ligand–receptor interaction types derived from the CellChatDB.human database, including secreted signaling, non-protein signaling, cell–cell contact, and ECM–receptor interactions. **(B)** Number of inferred interactions between different cell types. **(C)** Interaction strength between cell types based on aggregated communication probabilities. **(D)** Representative ligand–receptor signaling pathways mediating intercellular communication, including MIF–CD74–CXCR4 and PPIA–BSG interactions. **(E)** Global overview of inferred cell–cell communication patterns across all cell types.

Single-cell RNA-sequencing data from GSE149655 were analyzed, including two normal lung samples (GSM4506698, GSM4506700) and two LUAD samples (GSM4506699, GSM4506701). Following quality control and normalization, highly variable genes were identified, with the top 1,500 genes showing high coefficients of variation across cells. The ten most variable genes (IGLC2, HBA2, HBB, IGHG2, BPIFB1, HBA1, KRT17, SFRP2, COL3A1, and IGHG1) are shown in **Figure 1A**. Quality metrics including sequencing depth, detected gene number, and mitochondrial gene content are summarized in **Figure 1B**. After quality control, 6,931 single cells were retained, spanning seven major cell types with distinct distributions between tumor and adjacent normal tissues (**Supplementary Table S1**). Sequencing depth was positively correlated with gene counts, whereas its correlation with mitochondrial content was not statistically significant (**Figure 1C**). As shown in **Figure 1D**, PCA of all cells from the four GSE149655 samples demonstrated a largely overlapping distribution across samples. Principal component analysis identified PCs 1–20 as statistically significant based on JackStraw testing (*P* < 0.05; **Figure 1E**). Unsupervised clustering and annotation revealed 18 cell clusters corresponding to seven major cell types (**Figures 1F, G**): epithelial cells (clusters 0, 3, 7, 10, 11, 14, 15), T cells (cluster 1), endothelial cells (clusters 2, 8, 12, 13), adipocytes (clusters 4, 16), macrophages (clusters 5, 9), monocytes (clusters 6, 17), and fibroblasts (cluster 18). The top feature genes contributing to the major principal components are shown in **Figures 1H, I**. The overall analytical workflow is summarized in **Figure 2**. **Figure 3A** presents the annotation distribution results of seven cell types (monocytes, macrophages, fibroblasts, adipocytes, endothelial cells, epithelial cells, T cells) in the normal group and the lung adenocarcinoma patient group. **Figure 3B** illustrates a heatmap of the most significantly differentially expressed genes among the 18 clusters. Cell–cell communication analysis using CellChat identified diverse ligand–receptor interaction categories, including secreted signaling (39.6%), non-protein signaling (30.7%), cell–cell contact (16.6%), and ECM–receptor interactions (13.1%) (**Figure 4A**). Interaction networks revealed that epithelial cells predominantly functioned as ligand-expressing cells, with strong signaling toward endothelial cells, fibroblasts, and adipocytes (**Figure 4B**). Endothelial cells acted as both ligands and receptors, displaying strong interactions with epithelial cells, fibroblasts, and adipocytes but weaker interactions with T cells and monocytes. Interaction strength across cell types is summarized in **Figure 4C**. Specific signaling pathways were identified, including MIF-mediated communication between epithelial cells or fibroblasts and T cells via CD74–CXCR4. In addition, PPIA–BSG signaling was observed from endothelial cells to epithelial cells, adipocytes, fibroblasts, and endothelial cells themselves (**Figure 4D**). Global patterns of intercellular communication are illustrated in **Figure 4E**.

### Identification of endothelial cell–associated gene modules by hdWGCNA

**Figure 5:**
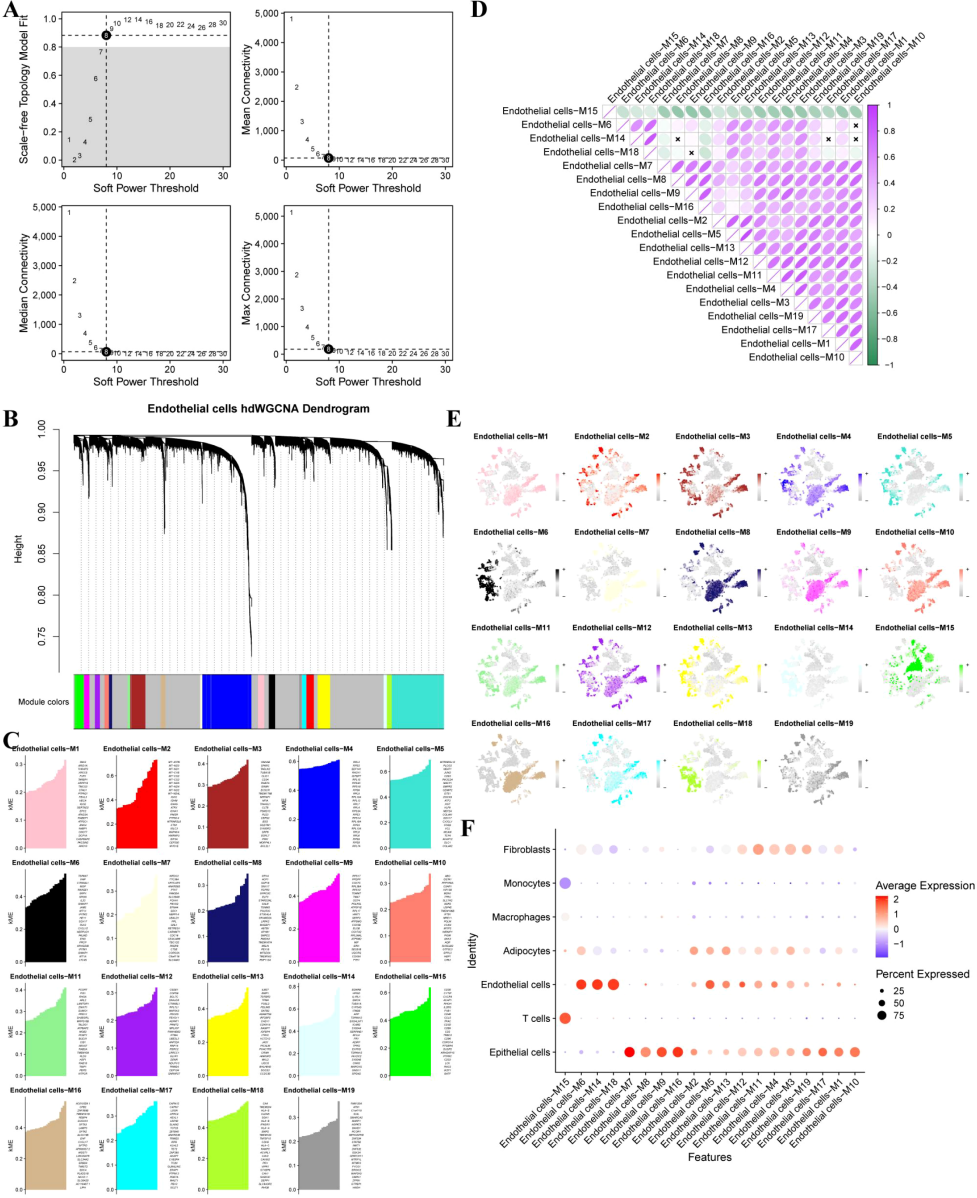
hdWGCNA analysis of endothelial cells. **(A)** Determination of the optimal Soft-thresholding power for hdWGCNA based on scale-free topology criteria. **(B)** Hierarchical clustering of endothelial cell–associated genes into co-expression modules. **(C)** Visualization of module eigengene–based connectivity (kME) for the top 25 hub genes in each module. **(D)** Correlation analysis among the identified gene modules. **(E)** Scatter plots illustrating module-level expression characteristics. **(F)** Correlation analysis between gene modules and annotated cell types.

**Figure 6:**
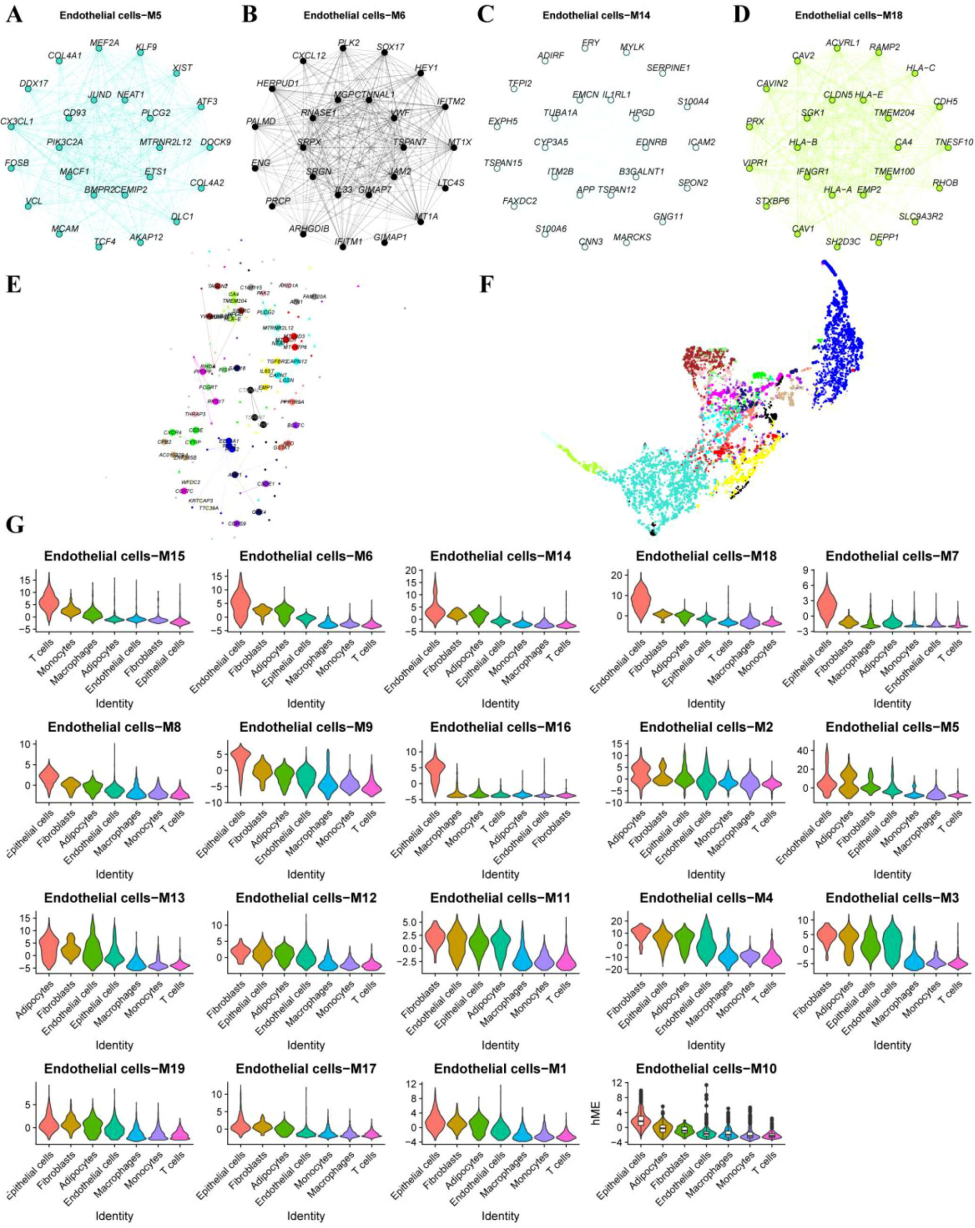
Identification of endothelial cell–associated core modules. **(A–D)** Regulatory network diagrams of core genes from four endothelial cell–associated modules. EIntegrated network of core genes across all identified modules. **(F)** Spatial distribution of module genes projected onto the UMAP embedding. **(G)** Violin plots showing module expression across major cell types.

High-dimensional weighted gene co-expression network analysis (hdWGCNA) was performed on endothelial cells. Soft-threshold power analysis identified power = 8 as optimal based on scale-free topology and average connectivity (**Figure 5A**). Using this parameter, a signed co-expression network was constructed, resulting in the identification of 19 distinct gene modules (**Figure 5B**). For each module, the topological overlap matrix (TOM) and module eigengenes were calculated, and the top 25 hub genes per module were visualized based on kME values (**Figure 5C**). Inter-module correlation analysis revealed a significant positive association between Module 6 and Modules 14 and 18 (**Figure 5D**). Module-wise expression distributions across endothelial cells are shown in **Figure 5E**. Comparison with single-cell annotations demonstrated that Modules 5, 6, 14, and 18 were preferentially expressed in endothelial cells. This endothelial enrichment was further confirmed by module–cell type correlation analysis (**Figure 5F**). Core gene networks of endothelial-associated modules are shown in **Figures 6A–D**, with an integrated network of all core genes presented in **Figure 6E**. Spatial distribution of module genes in UMAP space is shown in **Figure 6F**, and violin plots illustrating module expression across major cell types are shown in **Figure 6G**.

### Functional enrichment of EC-associated modules

**Figure 7:**
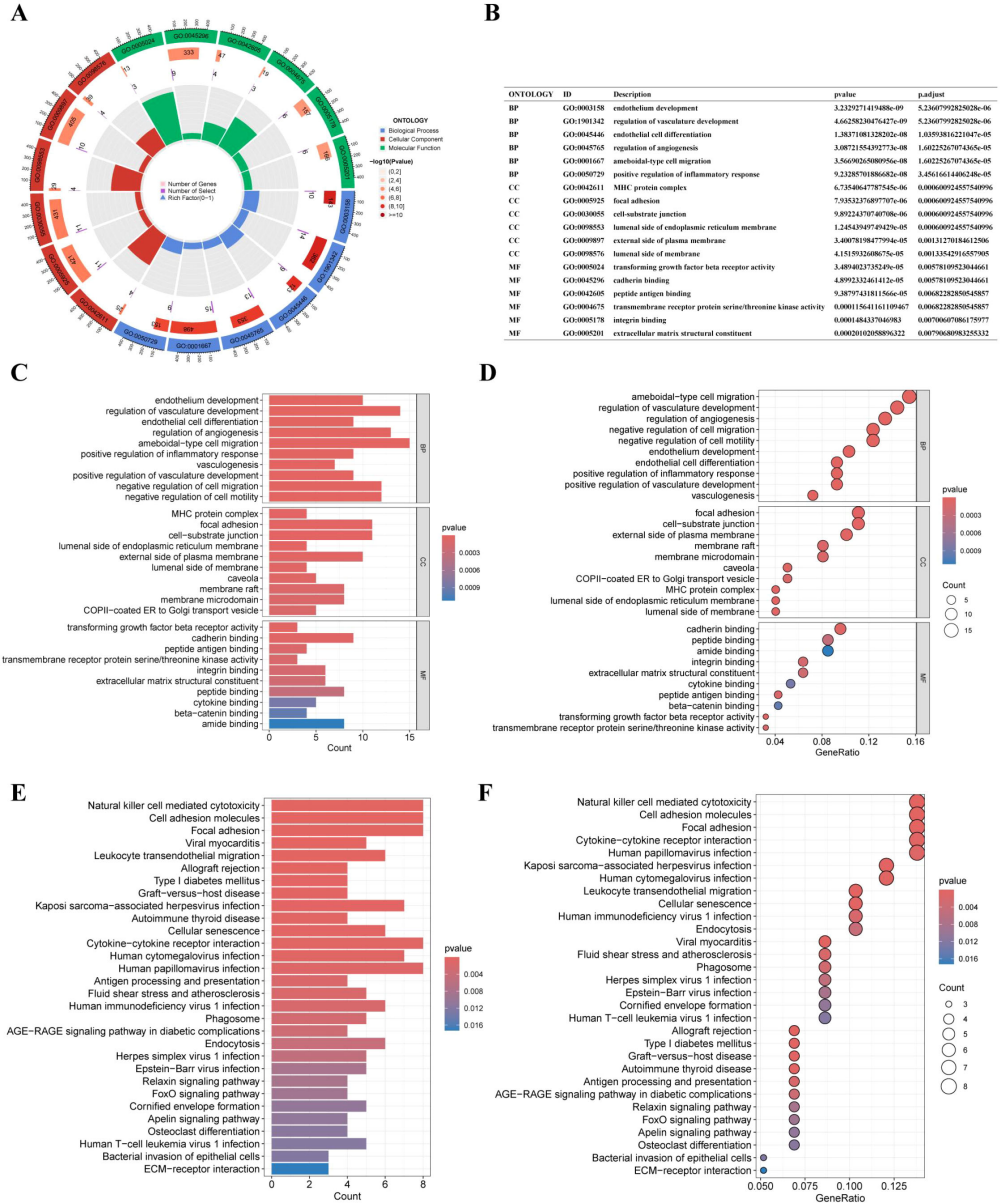
Functional enrichment analysis of endothelial-associated module genes. **(A, B)** Circular plots summarizing Gene Ontology (GO) enrichment of endothelial-associated module genes. **(C, D)** Bar plots and bubble plots displaying the top enriched GO terms across Biological Process, Cellular Component, and Molecular Function categories. **(E, F)** Bar plots and bubble plots of significantly enriched KEGG pathways.

Gene Ontology (GO) and KEGG pathway enrichment analyses were performed on endothelial-associated module genes. GO analysis revealed significant enrichment in endothelial cell differentiation (GO:0045446), MHC protein complex (GO:0042611), lumenal side of the endoplasmic reticulum membrane (GO:0098553), transforming growth factor beta receptor activity (GO:0005024), and transmembrane receptor protein serine/threonine kinase activity (GO:0004675) (**Figures 7A–D**). KEGG pathway analysis demonstrated enrichment in natural killer cell–mediated cytotoxicity, cell adhesion molecules, and focal adhesion pathways (**Figures 7E, F**).

### Differential expression of EC module genes in LUAD

**Figure 8:**
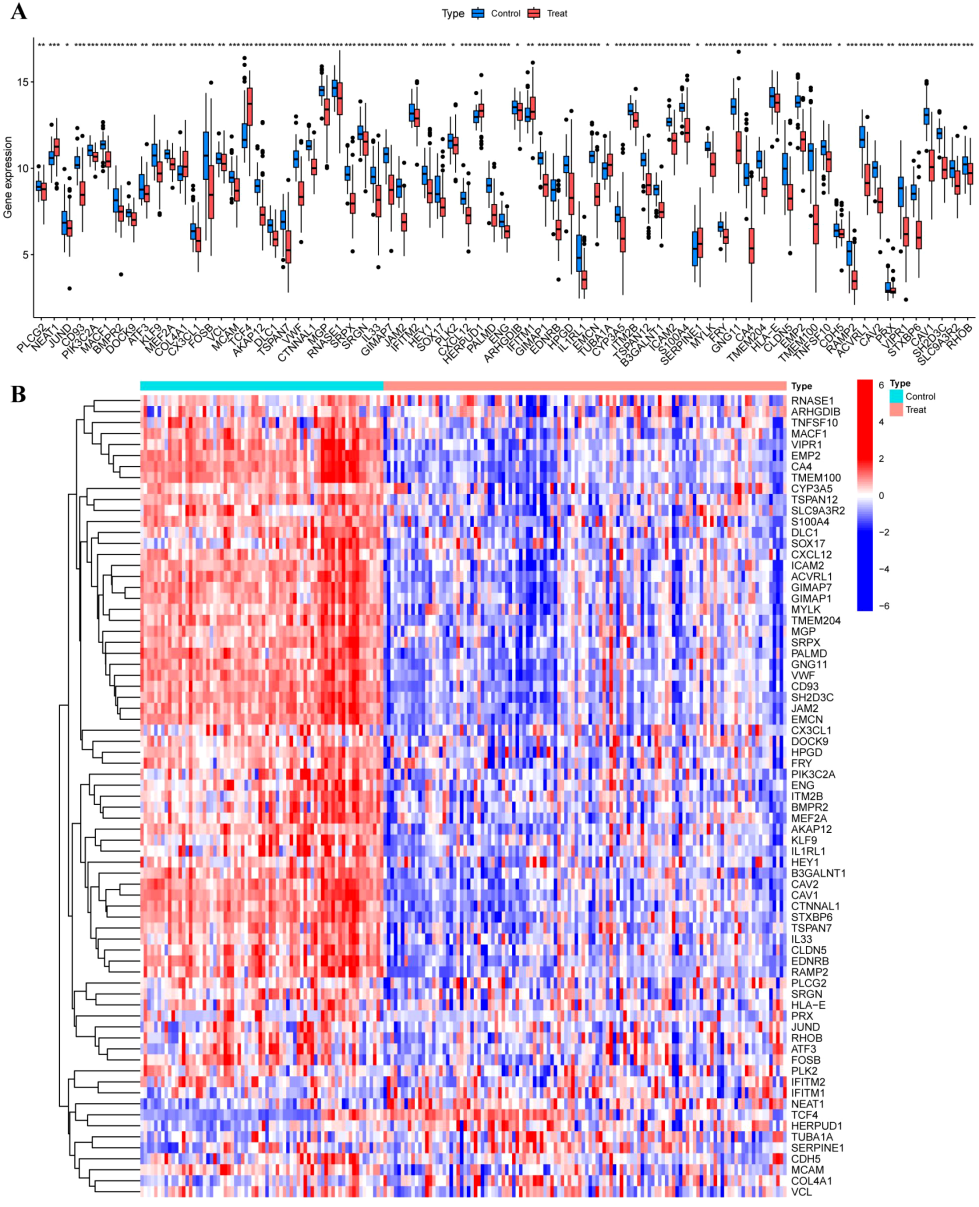
Differential expression analysis of endothelial-associated module genes in bulk cohorts. **(A)** Volcano plot showing differential expression of endothelial-associated module genes between LUAD and normal lung tissues across merged GEO datasets (GSE140797, GSE117049, GSE115002). **(B)** Heatmap illustrating expression patterns of endothelial-associated module genes across bulk samples.

Differential expression analysis of EC-associated module genes in bulk transcriptomic cohorts identified 73 genes significantly altered between normal and LUAD samples. Among these, NEAT1, TCF4, and IFITM1 were significantly upregulated in LUAD, whereas the remaining 70 genes exhibited reduced expression relative to normal tissues (**Figures 8A, B**).

### Gene-level discriminative ability of EC-related genes

**Figure 9:**
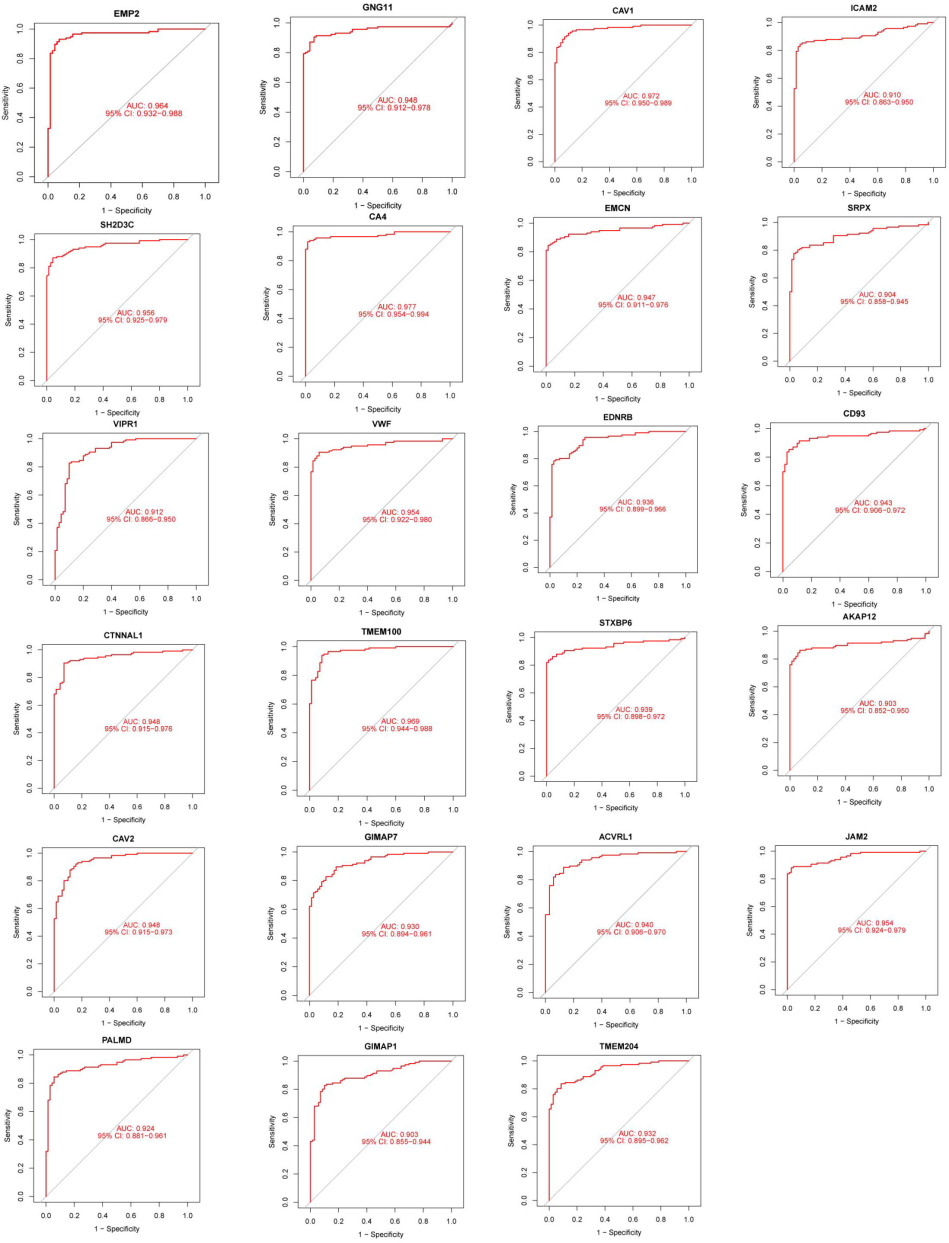
Gene-level ROC analysis of endothelial-associated module genes. Receiver operating characteristic (ROC) curves for 23 endothelial-associated genes exhibiting high discriminative performance (AUC > 0.9) in the discovery cohort. These analyses reflect gene-level discriminative ability rather than performance of a multigene diagnostic classifier.

Receiver operating characteristic (ROC) analysis was performed to assess the gene-level ability of differentially expressed EC-related genes to distinguish LUAD from normal samples in the discovery cohort. Twenty-three genes showed high apparent AUC values (>0.9) under this exploratory analysis (**Figure 9**). These results describe individual gene-level discrimination rather than the performance of a multi-gene diagnostic classifier and are not intended to indicate clinical diagnostic utility.

### Gene-level external validation in an independent cohort

The 23 EC-related genes identified in the discovery cohort were evaluated at the gene level in an independent bulk RNA-seq dataset (GSE85841) using the same ROC-based approach, without feature re-selection, refitting, or threshold optimization. Among these genes, 9 retained relatively high AUC values (>0.9), while the remaining genes showed attenuated discrimination (**Supplementary Figure S1**). These results support partial cross-cohort consistency at the individual-gene level rather than generalizable diagnostic performance.

### Exploratory machine learning–based prioritization of EC-associated genes

**Figure 10:**
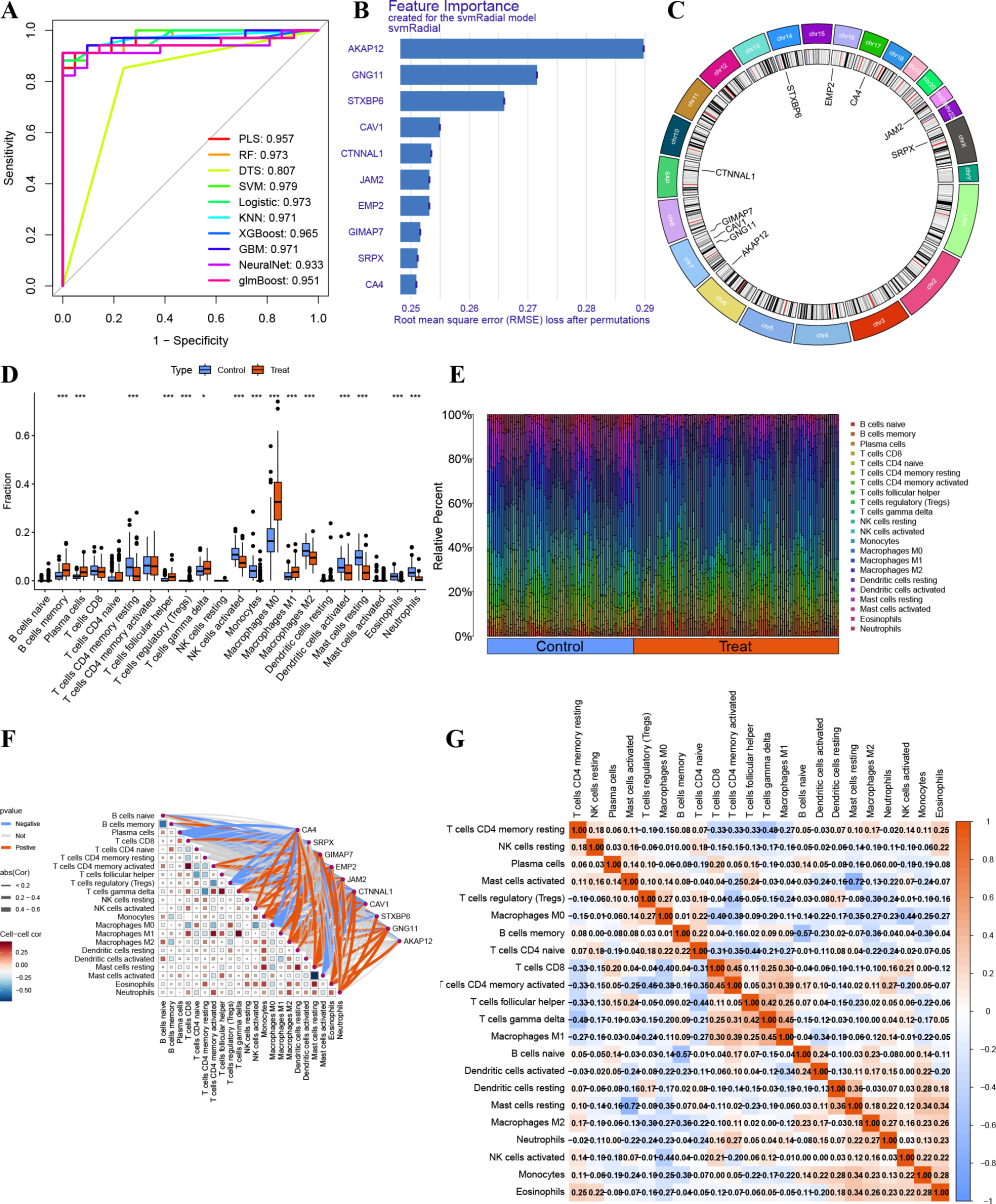
Exploratory machine learning–based gene prioritization and immune correlation analysis. **(A)** ROC curves comparing ten machine-learning algorithms based on a single train–test split within the discovery cohort. Curves reflect sample-level predictions on the held-out test set and are shown for exploratory model comparison rather than performance estimation. **(B)** Bar plot showing the top ten genes prioritized by feature importance in the SVM model. **(C)** Circular plot depicting chromosomal locations of the prioritized genes. **(D)** Differential abundance of 22 immune cell types between normal and LUAD samples. **(E)** Distribution of immune cell fractions across normal and LUAD groups. **(F)** Correlation analysis between prioritized genes and inferred immune cell fractions. **(G)** Correlation matrix showing relationships among immune cell types. Machine learning analyses were performed for exploratory gene prioritization and should not be interpreted as evidence of clinical diagnostic accuracy.

Machine learning analyses were performed on EC-associated genes using a single random train–test split. Among the ten evaluated algorithms, SVM showed the highest apparent AUC in the test set under this exploratory setup (**Figure 10A**). This model was therefore used as a ranking tool to prioritize EC-associated genes for downstream analysis rather than as a diagnostic classifier. Gene importance scores derived from SVM were used to rank features, and the top ten EC-associated genes were highlighted (**Figure 10B**), with their chromosomal locations shown in **Figure 10C**. These results indicate that, under the present exploratory conditions, SVM provided a convenient framework for relative feature prioritization. Importantly, ML-based AUC values are reported only to illustrate relative model behavior rather than diagnostic accuracy, and all biological interpretations in this study are based on gene-level consistency across datasets and independent functional validation, rather than on classifier performance.

### Immune infiltration and correlation analyses

**Figure 11:**
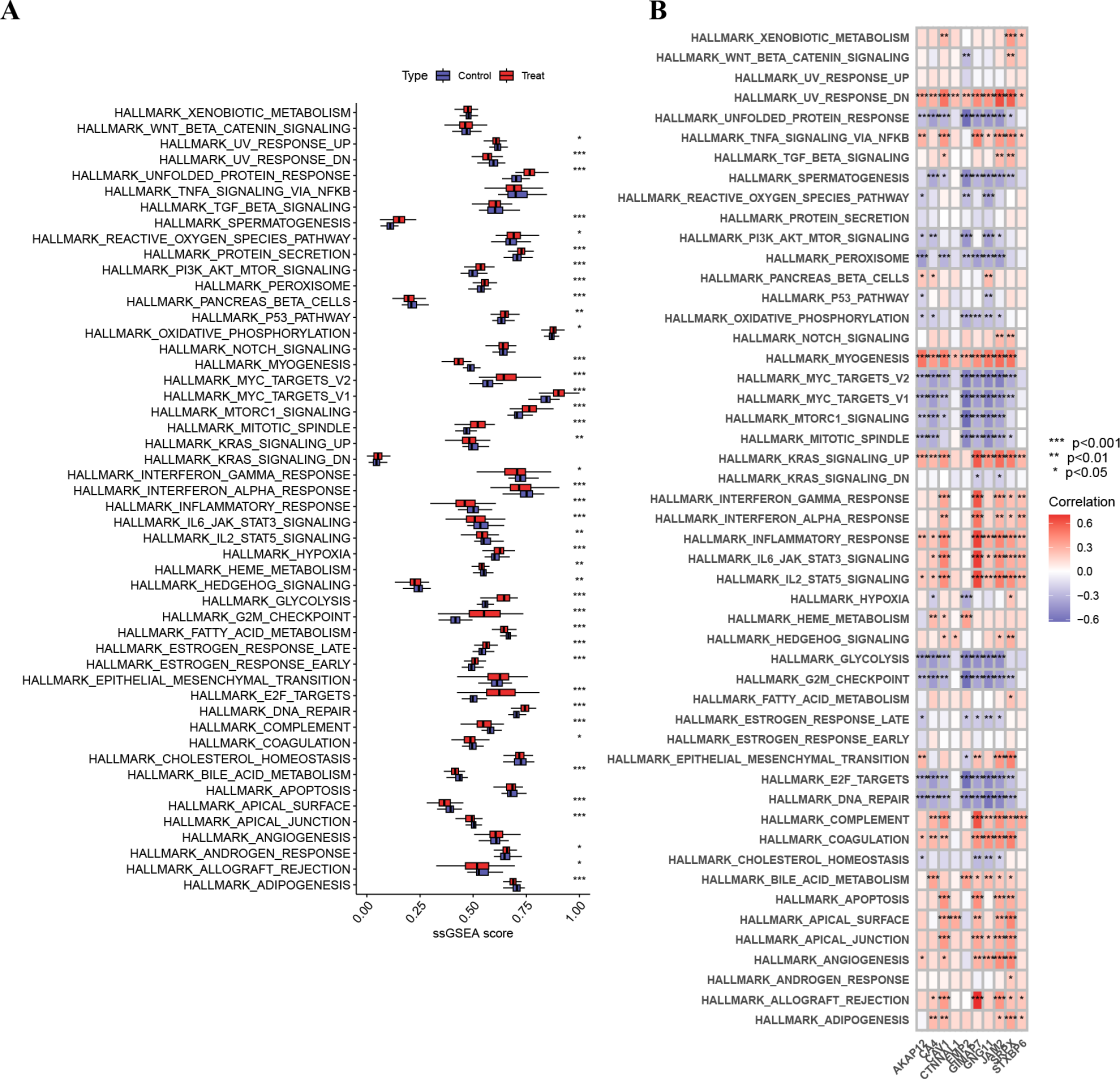
Single-sample GSEA and pathway correlation analysis. **(A)** Differential analysis of ssGSEA pathway scores between normal and LUAD samples. **(B)** Correlation analysis between prioritized genes and KEGG pathway enrichment scores.

Immune cell deconvolution revealed significant differences in the abundance of multiple immune cell types between normal and LUAD samples, including memory B cells, plasma cells, CD4 memory T cells, follicular helper T cells, regulatory T cells, γδ T cells, activated NK cells, monocytes, macrophage subsets, activated dendritic cells, mast cells, eosinophils, and neutrophils (**Figure 10D**). Macrophage M0 abundance was notably increased in LUAD samples. Immune cell distributions and intercellular correlations are shown in **Figures 10E–G**. Single-sample GSEA analysis of hallmark pathways identified 28 KEGG pathways with significant differences between normal and LUAD samples (*P* < 0.001; **Figure 11A**). Correlation analysis revealed positive associations between GIMAP7 and inflammatory response–related pathways, whereas EMP2 was negatively correlated with cell cycle–associated pathways, including E2F targets, G2M checkpoint, and mTORC1 signaling (**Figure 11B**).

### Network pharmacology prioritizes MYLK as an endothelial-associated candidate gene

**Figure 12:**
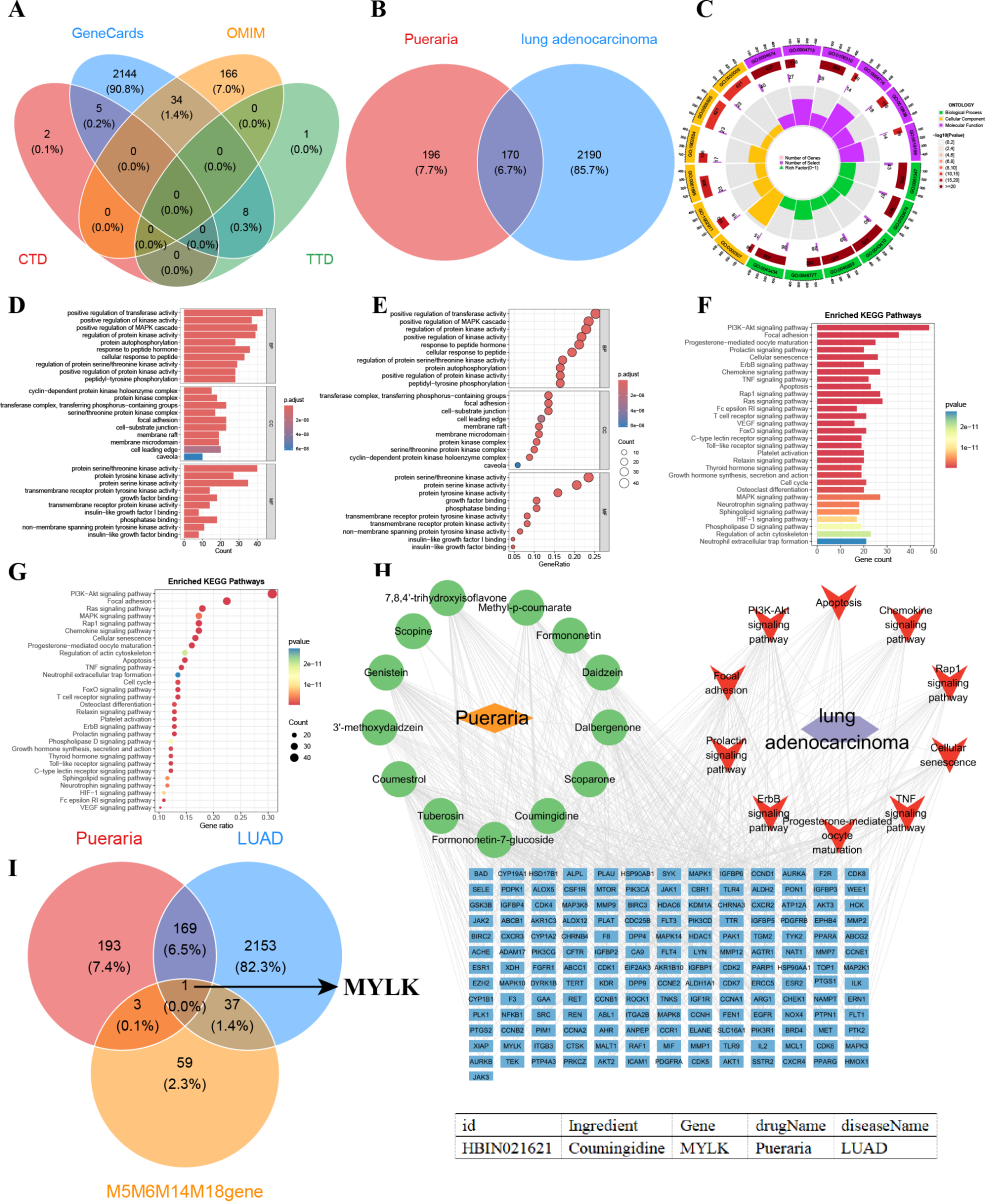
Network pharmacology analysis identifying MYLK as an endothelial-associated candidate gene. **(A)** Compilation of LUAD-related genes from the CTD, OMIM, TTD, and GeneCards databases. **(B)** Intersection of LUAD-related genes with predicted targets of Pueraria-derived compounds. **(C–E)** GO enrichment analysis of intersecting genes. **(F, G)** KEGG pathway enrichment analysis of intersecting genes. **(H)** Integrated regulatory network comprising intersecting genes, Pueraria-derived compounds, LUAD, and enriched KEGG pathways. **(I, J)** Intersection of compound-associated genes, LUAD-related genes, and endothelial-associated gene modules (M5, M6, M14, M18), highlighting MYLK as a candidate gene identified by in silico analyses.

A total of 2360 LUAD-related genes obtained from the CTD, TTD, OMIM, and Genecards databases are shown in **Figure 12A**. Intersection analysis between LUAD-related genes and a herb-derived compound target library identified 170 overlapping genes (**Figure 12B**). GO enrichment of these genes showed significant enrichment in protein serine/threonine kinase activity and regulation of transferase activity (**Figures 12C–E**), while KEGG analysis highlighted PI3K–Akt signaling and focal adhesion pathways (**Figures 12F, G**). An integrated regulatory network comprising Pueraria, its 13 active compounds, LUAD, enriched KEGG pathways, and overlapping genes was constructed (**Figure 12H**). Intersection of these genes with EC-associated modules (M5, M6, M14, M18) identified MYLK as an endothelial-associated candidate gene emerging from network pharmacology, with in silico analyses suggesting potential small-molecule compatibility (**Figures 12I, J**). Molecular docking and molecular dynamics simulations suggested a stable MYLK–Coumingidine interaction (**Supplementary Figure S2**).

### MYLK expression and prognostic relevance in LUAD

**Figure 13:**
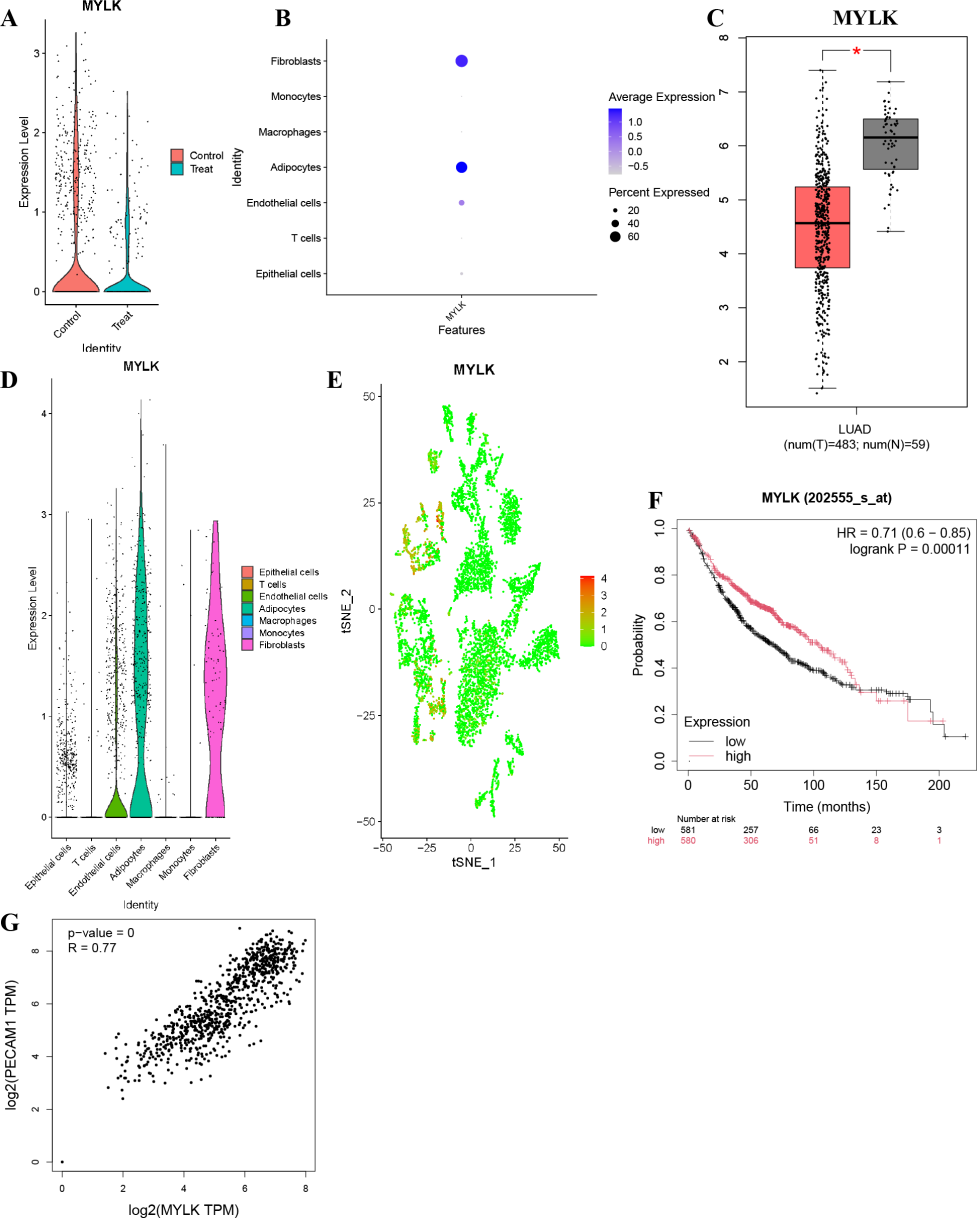
MYLK expression patterns and prognostic relevance in LUAD. **(A)** MYLK expression in endothelial cells from adjacent non-malignant and LUAD tissues. **(B)** MYLK expression across major stromal and immune cell types. **(C)** GEPIA analysis comparing MYLK expression between LUAD and normal lung tissues. **(D)** Violin plots showing MYLK expression across annotated cell types. **(E)** Scatter plot of MYLK expression distribution in the GSE149655 single-cell dataset. **(F)** Kaplan–Meier survival analysis of MYLK expression in LUAD patients. **(G)** Correlation analysis between MYLK and PECAM1 (CD31) expression.

MYLK expression was significantly higher in endothelial cells from adjacent non-malignant tissues compared with tumor tissues (**Figure 13A**). Elevated MYLK expression was also observed in fibroblasts and adipocytes, whereas reduced expression was detected in T cells, monocytes, and macrophages (**Figures 13B, D**). Scatter plot analysis confirmed higher MYLK expression in endothelial cells from non-tumor tissues (**Figure 13E**). Analysis using GEPIA revealed significantly lower MYLK expression in LUAD tissues compared with normal lung tissues (**Figure 13C**), and reduced MYLK expression was associated with unfavorable patient prognosis (**Figure 13F**). MYLK expression showed a strong positive correlation with PECAM1 (CD31) expression (R = 0.77, *P* < 0.001; **Figure 13G**).

### Endothelial functional validation of MYLK

**Figure 14:**
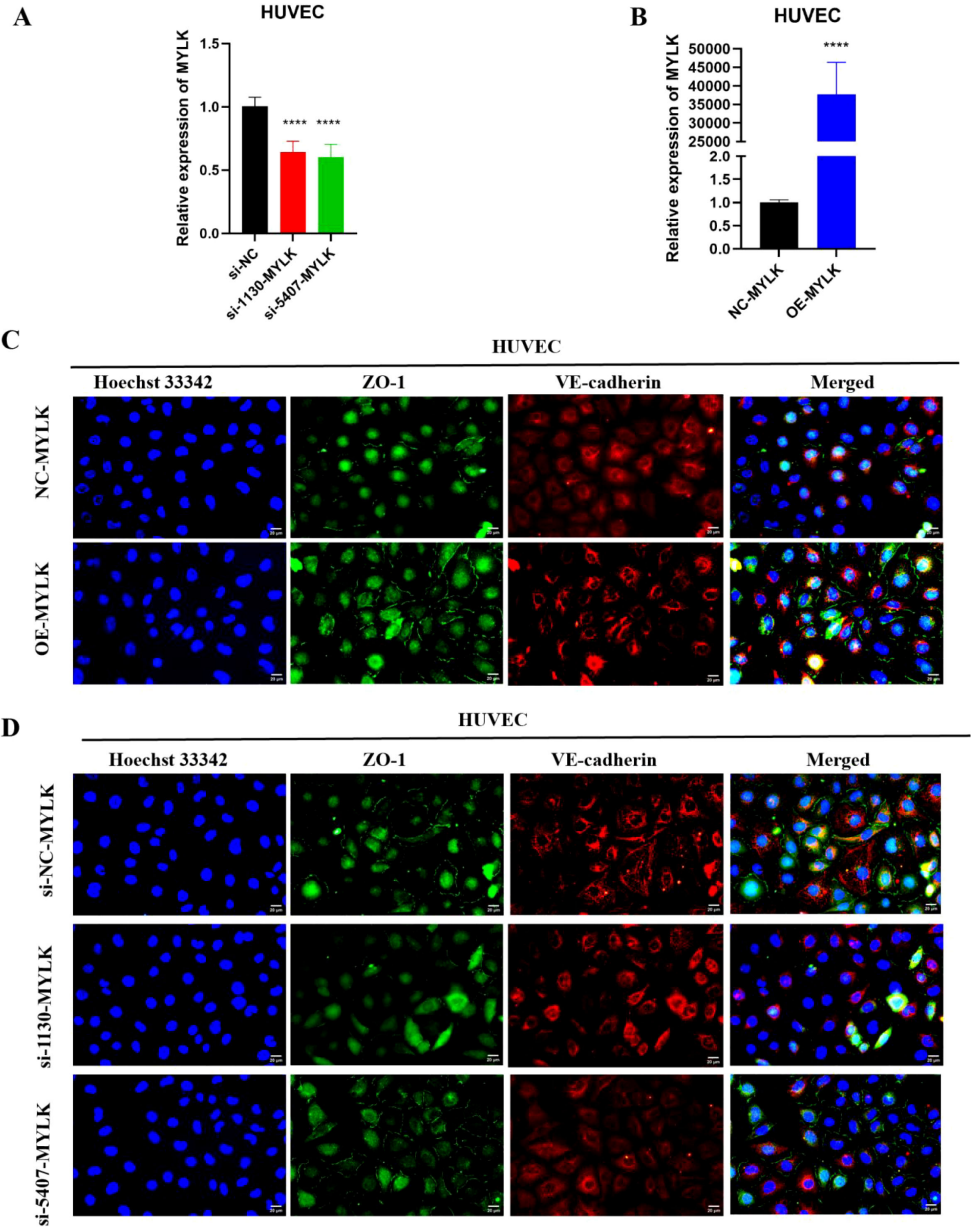
MYLK modulates endothelial junction organization in HUVECs. **(A, B)** RT-qPCR analysis confirming MYLK overexpression and knockdown in HUVECs. **(C)** Representative immunofluorescence images of HUVECs transfected with NC-MYLK or OE-MYLK, stained for ZO-1 (green), VE-cadherin (red), and nuclei (Hoechst 33342, blue). MYLK overexpression enhanced continuous junctional localization of ZO-1 and VE-cadherin. **(D)** Representative immunofluorescence images of HUVECs transfected with si-NC, si-1130-MYLK, or si-5407-MYLK. MYLK knockdown disrupted junctional organization, as indicated by reduced and discontinuous ZO-1 and VE-cadherin staining. Images were acquired under identical exposure settings. Scale bar: 20 μm. **** indicates P < 0.0001.

**Figure 15:**
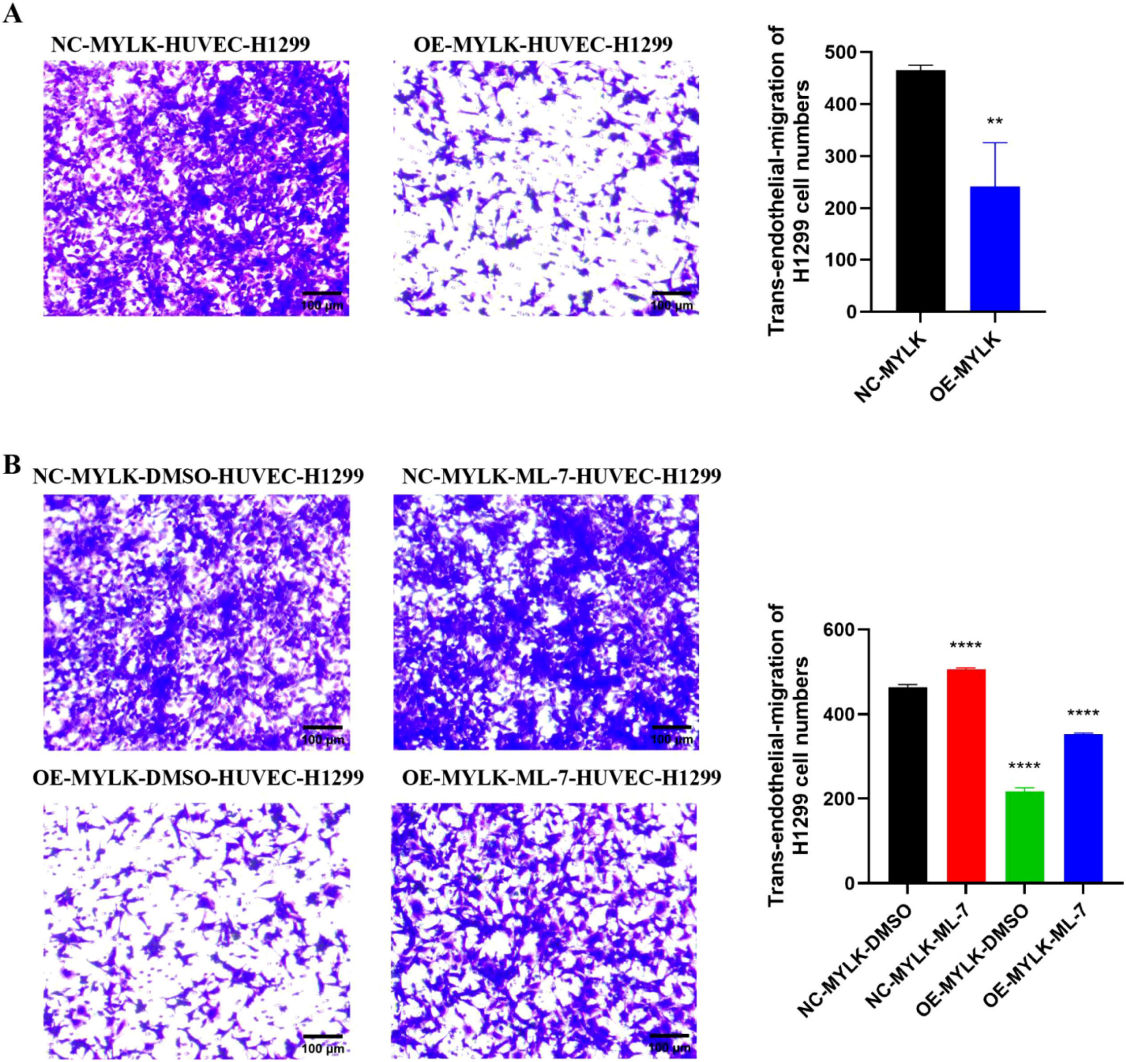
MYLK influences endothelial barrier properties in a trans-endothelial migration assay. **(A)** Representative images and quantification of H1299 cell transmigration across HUVEC monolayers transfected with control or MYLK-overexpression constructs. **(B)** Effects of ML-7 pretreatment on trans-endothelial migration, showing increased tumor cell transmigration and partial attenuation of MYLK-associated barrier enhancement. Data are presented as mean ± SD from three independent experiments. Scale bar: 100 μm. ** indicates P < 0.01 and **** indicates P < 0.0001.

MYLK expression was efficiently modulated in HUVECs using overexpression and siRNA-mediated knockdown strategies (**Figures 14A, B**). Immunofluorescence analysis enhanced junctional localization of VE-cadherin and ZO-1 in MYLK-overexpressing endothelial monolayers, whereas MYLK knockdown disrupted junctional continuity (**Figures 14C, D**). In trans-endothelial migration assays, MYLK overexpression in HUVECs significantly reduced H1299 tumor cell transmigration compared with controls (**Figure 15A**). Pharmacological inhibition with ML-7 increased tumor cell transmigration and partially reversed the barrier-protective effects of MYLK overexpression (**Figure 15B**). These findings indicate that MYLK contributes to endothelial barrier integrity in this experimental setting.

### Tumor cell-intrinsic effects of MYLK

**Figure 16:**
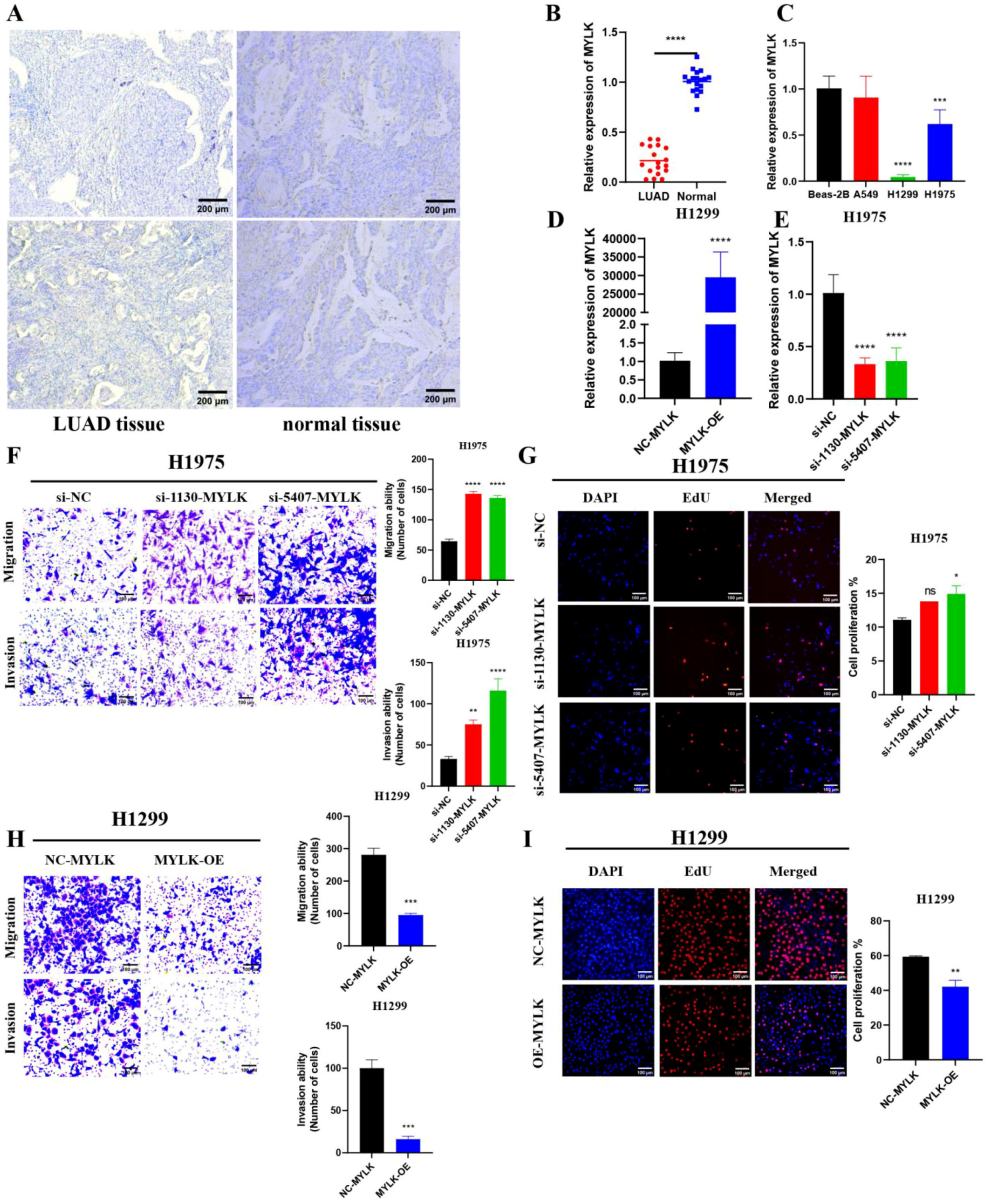
Tumor cell-intrinsic effects of MYLK in LUAD. **(A)** Representative IHC staining of MYLK in LUAD tissues and matched adjacent normal lung tissues. **(B)** Relative MYLK mRNA expression in paired LUAD and adjacent normal tissues determined by RT-qPCR. C MYLK expression levels in LUAD cell lines compared with Beas-2B cells. **(C)** Relative expression of MYLK in Beas-2B, A549, H1299 and H1975. **(D, E)** Validation of MYLK overexpression and knockdown in LUAD cell lines using RT-qPCR. **(F, H)** Migration and invasion assays following MYLK knockdown or overexpression. **(G, I)** EdU proliferation assays showing changes in tumor cell proliferation after MYLK modulation. Data are presented as mean ± SD from three independent experiments. Scale bar: 200 μm **(A)**; 100 μm **(F–I)**. ns indicates no statistically significant difference, * indicates P < 0.05, ** indicates P < 0.01, *** indicates P < 0.001, and **** indicates P < 0.0001.

Representative IHC images showed reduced MYLK protein expression in LUAD tissues compared with matched adjacent normal lung tissues (**Figure 16A**). Consistently, RT-qPCR analysis of paired clinical samples demonstrated lower MYLK mRNA levels in tumor tissues than in adjacent normal controls (**Figure 16B**). MYLK expression was also decreased in LUAD cell lines relative to Beas-2B cells (**Figure 16C**). Functional assays demonstrated that MYLK knockdown in H1975 cells increased migration, invasion, and proliferation, whereas MYLK overexpression in H1299 cells suppressed these malignant phenotypes (**Figures 16D–I**).

### Network-based in silico knockout of MYLK supports endothelial-centered transcriptional programs

In addition to experimental perturbation of MYLK in endothelial and tumor cell models, we performed a network-based in silico knockout analysis using scTenifoldKnk in independent tumor samples. Virtual removal of MYLK regulatory influence resulted in consistent perturbations enriched in endothelial-associated biological processes, including endothelial apoptosis, tight junction organization, angiogenesis, vascular development, and regulation of vascular permeability (**Supplementary Figures S3A–F**). These results indicate convergence of MYLK-associated regulatory networks on endothelial-related transcriptional programs.

### Cell-type–specific associations of MYLK in tumor cells and dendritic cells

**Figure 17:**
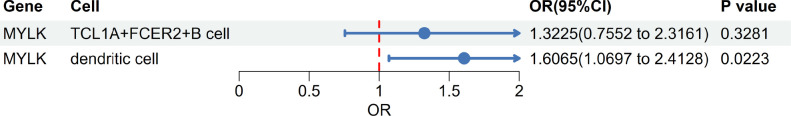
Cell-type–specific genetic association of MYLK in LUAD. Forest plot showing the association between MYLK-related genetic variants in dendritic cells and LUAD risk based on single-cell eQTL analysis. OR > 1 indicates increased risk; *P* = 0.0223.

Single-cell eQTL analysis using ONEK1K data identified MYLK-associated genetic signals in four immune cell types, including NK cells, monocytes, dendritic cells, and BIN B cells. Among these, alleles associated with higher MYLK expression (or genetically predicted activity proxy) in dendritic cells were significantly associated with elevated LUAD risk (OR = 1.6065, *P* = 0.0223; **Figure 17**). These findings suggest the presence of cell-type-specific genetic associations involving MYLK in dendritic cells; however, they do not imply causality or functional involvement and are intended solely to generate hypotheses for future studies.

## Discussion

Lung adenocarcinoma (LUAD) is a highly heterogeneous malignancy whose progression is strongly influenced by the tumor microenvironment. Beyond malignant epithelial cells, stromal components—particularly endothelial cells (ECs)—play central roles in angiogenesis, vascular permeability, immune cell trafficking, and tumor–immune interactions. Despite advances in targeted and immune-based therapies, the endothelial-associated regulatory mechanisms shaping LUAD progression remain incompletely defined (27–30).

In this study, we employed an integrated single-cell and network-based framework to characterize endothelial-centered transcriptional programs in LUAD. By combining scRNA-seq analysis with hdWGCNA, we identified EC-associated gene modules that were not readily captured by bulk transcriptomic approaches. Doublet detection was not explicitly performed due to the limited availability of matched cell hashing or genetic demultiplexing information. Consistent with prior applications of hdWGCNA in metastatic macrophage subpopulations and CNV-defined malignant cell populations across cancer types, our results support the utility of network-based methods for uncovering coordinated regulatory programs within specific cellular compartments of the tumor microenvironment (31, 32). Importantly, the EC-associated modules identified here were enriched in pathways related to junction organization, angiogenesis, and immune regulation, supporting the concept that endothelial dysfunction extends beyond vascular remodeling to broader microenvironmental regulation.

Cell–cell communication analysis further revealed extensive interactions between ECs and epithelial cells, fibroblasts, adipocytes, and immune cells. Epithelial cells predominantly acted as ligand-expressing populations, whereas ECs functioned as both signal senders and receivers, consistent with their central coordinating role within the tumor microenvironment (33–36). These findings suggest that endothelial–epithelial crosstalk may contribute to immune modulation and structural remodeling in LUAD, although functional validation of specific ligand–receptor axes will be required in future studies.

It should be emphasized that the machine-learning analyses in this study were conducted solely to compare relative model behavior and to support feature prioritization for downstream analysis, rather than to develop or evaluate diagnostic classifiers. Multiple machine-learning algorithms were evaluated using a single random train-test split, under which SVM exhibited the highest apparent AUC in the test set. This model was therefore used as a ranking tool to prioritize EC-associated genes for further analysis, rather than as evidence of predictive superiority. This exploratory approach did not involve group-wise splitting, nested cross-validation, or fold-internal preprocessing, and therefore the resulting performance metrics should not be interpreted as estimates of generalizable diagnostic accuracy. Future studies incorporating rigorous validation frameworks will be required to assess any potential clinical applicability of these findings.

Through integration of EC-associated modules with network pharmacology analysis, myosin light chain kinase (MYLK) was prioritized as a candidate gene linking endothelial regulation with LUAD-related pathways. MYLK is known to regulate cytoskeletal dynamics, junctional stability, and contractility in endothelial cells, processes critical for vascular integrity and permeability (37–41). While MYLK has been studied in vascular biology and inflammatory contexts, its functional relevance in LUAD, particularly within an endothelial-centered framework, has remained insufficiently characterized.

Consistent with the endothelial-centered discovery framework, we prioritized endothelial-specific perturbation and readouts as the primary experimental validation strategy. Experimental perturbation of MYLK in endothelial cells demonstrated that MYLK gain- and loss-of-function directly modulated junctional organization and trans-endothelial tumor cell migration, consistent with a role for MYLK-associated signaling in endothelial barrier regulation. While the trans-endothelial migration assay was critical to our investigation of endothelial barrier function, we acknowledge that the lack of definitive tumor cell labeling may limit the interpretation of these results. Specifically, although tumor cells were used in the assay, the absence of distinct tumor-specific markers in the endothelial monolayer makes it difficult to conclusively rule out the migration of non-tumor cells. Future studies incorporating cell-specific labeling and tracking techniques will be needed to fully validate the tumor-endothelial interaction dynamics. ScTenifoldKnk provides a network-based framework to assess the regulatory consequences of virtual gene perturbation from single-cell data, capturing pathway-level rewiring beyond differential expression (42). In this study, it was used as a complementary, hypothesis-generating approach to evaluate whether MYLK-associated effects converge on endothelial-related programs in independent tumor datasets, rather than as a substitute for genetic knockout or causal inference. These endothelial-specific findings were complemented by network-based in silico knockout analyses performed in independent LUAD tumor samples, which revealed transcriptional perturbations enriched for endothelial-associated biological processes, including tight junction organization, angiogenesis, vascular permeability, and endothelial apoptosis. Although this in silico approach does not establish cell-type–specific causality, it provides systems-level evidence that MYLK-associated regulatory networks converge on endothelial-centered programs within the tumor context.

In parallel, MYLK perturbation in LUAD cell lines revealed a distinct, tumor cell–intrinsic role. MYLK overexpression suppressed, whereas MYLK knockdown enhanced, tumor cell proliferation, migration, and invasion, consistent with a tumor-suppressive function in cancer cells. Together with the endothelial findings, these results highlight the context-dependent nature of MYLK activity and underscore the importance of cell-type specificity when interpreting its biological roles within the tumor microenvironment.

To further explore immune-related associations, we performed exploratory single-cell eQTL analyses, which suggested that increased MYLK-associated genetic signals in dendritic cells are statistically associated with elevated LUAD risk. These observations are associative in nature and do not establish causality. Rather than supporting a defined immune mechanism, they indicate that MYLK-related regulatory signals may differ across tumor, endothelial, and immune cell contexts. Importantly, the single-cell eQTL analysis in dendritic cells was performed for exploratory purposes only and does not support causal inference; no multiple-testing correction, colocalization with LUAD risk loci, or functional perturbation was conducted. Accordingly, these findings should be interpreted strictly as hypothesis-generating and are not incorporated into the core mechanistic framework of the present study.

Although molecular docking and molecular dynamics simulations suggested a potentially stable interaction between MYLK and the natural compound Coumingidine, this interaction was not experimentally validated in the present study due to limitations in compound availability and synthesis feasibility. Importantly, the present work does not propose Coumingidine as a validated MYLK inhibitor or a therapeutic candidate. Instead, network pharmacology and in silico docking were applied as exploratory approaches to contextualize MYLK within endothelial-associated regulatory pathways. Accordingly, ML-7 (43), a well-characterized MYLK inhibitor, was used as a pharmacological tool to probe MYLK-dependent endothelial functions rather than to assess Coumingidine-MYLK target engagement. ML-7 pretreatment partially modulated MYLK-associated endothelial phenotypes, supporting the involvement of MYLK-related signaling pathways in this experimental context. Future studies incorporating biochemical kinase assays, *in vivo* models, and cell-type-specific MYLK perturbation strategies will be required to establish definitive target engagement, causal mechanisms, and therapeutic relevance.

In conclusion, this integrated single-cell and network-based analysis delineates endothelial-associated transcriptional programs that contribute to LUAD progression and identifies MYLK as a context-dependent regulatory node within the tumor microenvironment. MYLK exhibits distinct roles across cellular compartments, influencing endothelial junction integrity, tumor cell behavior, and potentially immune-related processes. While network-based and computational analyses nominate MYLK as a candidate within endothelial-associated regulatory pathways, these findings are exploratory and do not establish therapeutic efficacy. Overall, this study provides a systems-level framework for understanding endothelial regulation in LUAD and offers a foundation for future mechanistic and *in vivo* investigations.

## Data Availability

Sequence data that support the findings of this study have been deposited in the Gene Expression Omnibus (GEO) database with the primary accession code PRJNA629546, PRJNA590980, PRJNA480904, PRJNA473511. The molecular docking data support the findings of this study was downloaded from CB-dock2 website. The Pueraria ingredients was downloaded from PubChem database. The structure of MYLK was downloaded from RSCB PDB database.

## Supplementary material

Supplementary Figure S1 External validation of endothelial cell–related genes in GSE85841. Gene-level ROC analysis of 23 pre-specified endothelial-associated genes in the independent GSE85841 dataset without feature re-selection. AUC attenuation relative to the discovery cohort is observed, with a subset of genes retaining high discriminative performance.

Supplementary Figure S2 Molecular docking and molecular dynamics (MD) simulations of the MYLK–Coumingidine complex. **(A)** Predicted docking pose of Coumingidine within the MYLK structure. **(B)** Root mean square deviation (RMSD) of the MYLK–Coumingidine complex over a 100-ns MD simulation. **(C)** Radius of gyration (Rg) analysis demonstrating overall structural compactness of the protein–ligand complex during simulation. **(D)** Solvent-accessible surface area (SASA) of the MYLK–Coumingidine complex over time. **(E)** Number of hydrogen bonds between MYLK and Coumingidine throughout the simulation. These simulations suggest a stable interaction between MYLK and Coumingidine; however, due to synthesis and availability constraints, this interaction was not experimentally validated and is presented as hypothesis-generating.

Supplementary Figure S3 Network-based in silico knockout of MYLK in tumor samples. **(A)** Volcano plot of genes affected by virtual MYLK knockout using scTenifoldKnk. **(B)** Top differentially regulated genes following MYLK network perturbation. **(C, D)** GO enrichment analyses of network-derived genes. **(E, F)** KEGG pathway enrichment analyses of network-derived genes.

Supplementary Table S1 Summary of single-cell RNA-seq quality control and cell composition. **(A)** Number of retained cells per sample (orig.ident) after QC filtering. **(B)** Number of retained cells per Seurat cluster (resolution = 0.6). **(C)** Total number of retained cells per annotated major cell type. **(D)** Distribution of annotated cell types across adjacent normal (Control) and tumor (Treat) tissues. E Total number of cells retained after QC filtering.
