# Supplementary figures and images for: An endothelial-centered regulatory framework reveals context-dependent roles of MYLK in lung adenocarcinoma
Source: Front Immunol. 2026 Apr 1;17:1719296. doi: 10.3389/fimmu.2026.1719296 (PMC13079312; doi:10.3389/fimmu.2026.1719296)

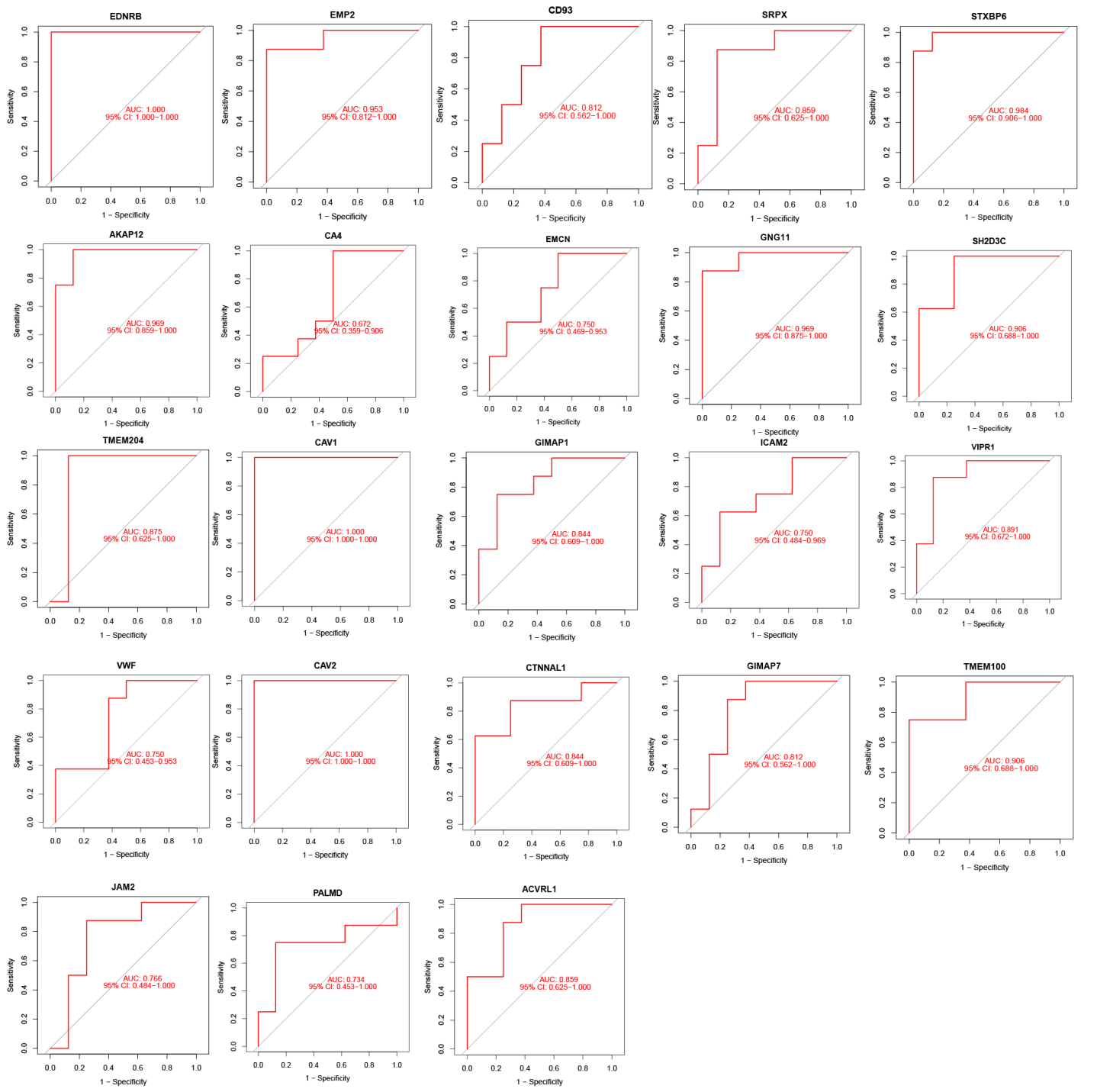

Supplement: Supplementary Figure S1 — External validation of endothelial cell–related genes in GSE85841. Gene-level ROC analysis of 23 pre-specified endothelial-associated genes in the independent GSE85841 dataset without feature re-selection. AUC attenuation relative to the discovery cohort is observed, with a subset of genes retaining high discriminative performance. [file Image1.tif]

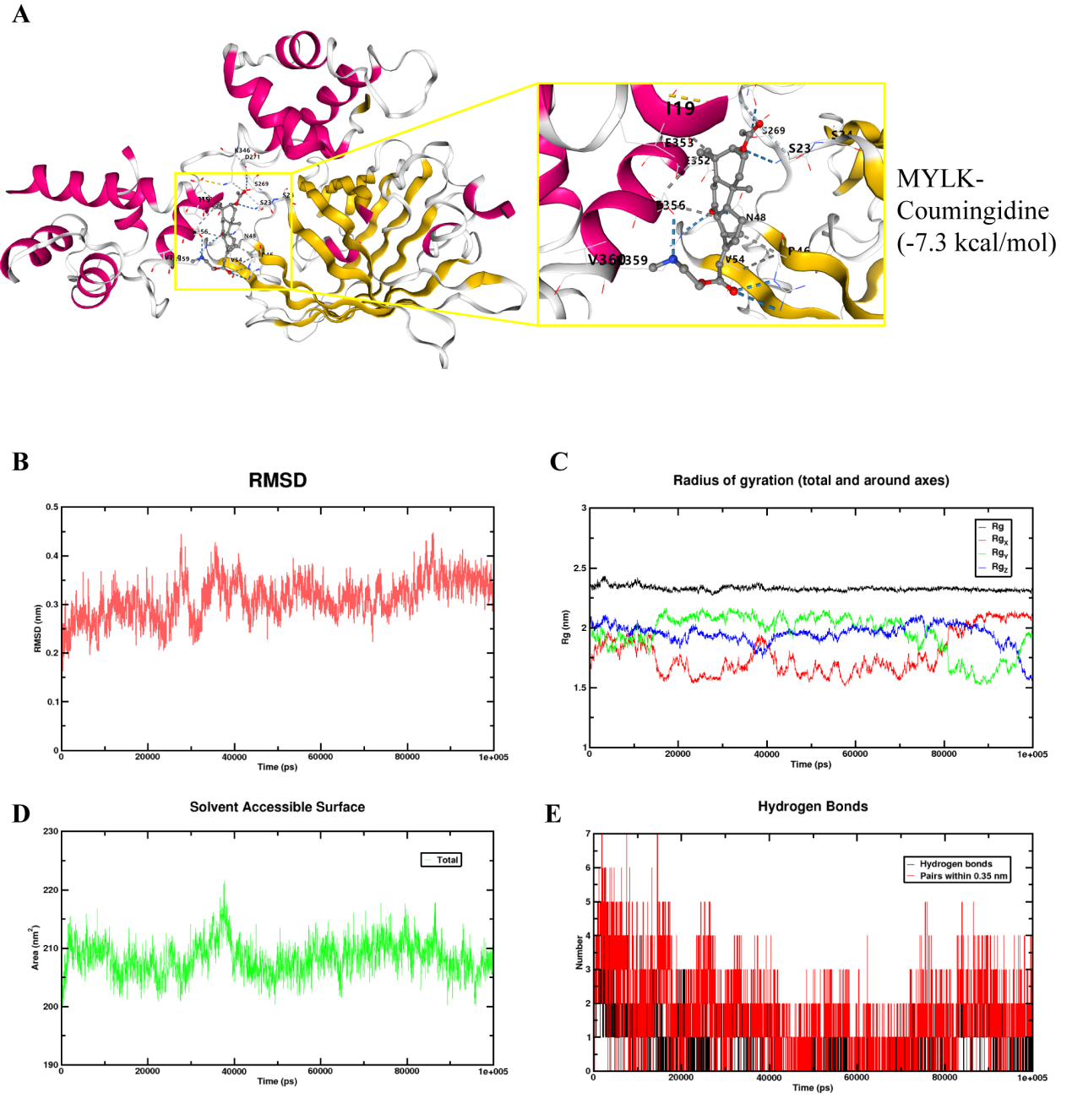

Supplement: Supplementary Figure S2 — Molecular docking and molecular dynamics (MD) simulations of the MYLK–Coumingidine complex. (A) Predicted docking pose of Coumingidine within the MYLK structure. (B) Root mean square deviation (RMSD) of the MYLK–Coumingidine complex over a 100-ns MD simulation. (C) Radius of gyration (Rg) analysis demonstrating overall structural compactness of the protein–ligand complex during simulation. (D) Solvent-accessible surface area (SASA) of the MYLK–Coumingidine complex over time. (E) Number of hydrogen bonds between MYLK and Coumingidine throughout the simulation. These simulations suggest a stable interaction between MYLK and Coumingidine; however, due to synthesis and availability constraints, this interaction was not experimentally validated and is presented as hypothesis-generating. [file Image2.tif]

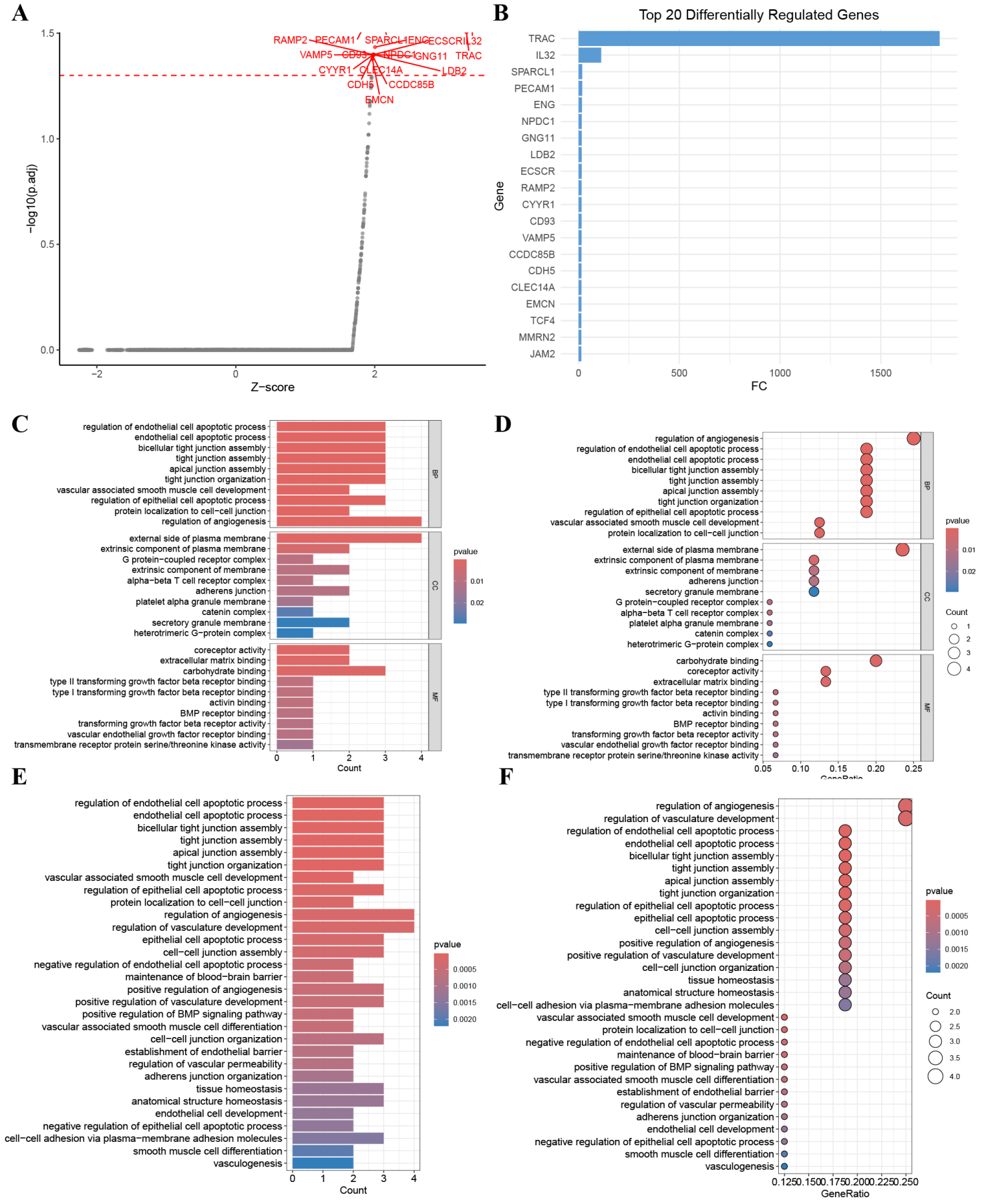

Supplement: Supplementary Figure S3 — Network-based in silico knockout of MYLK in tumor samples. (A) Volcano plot of genes affected by virtual MYLK knockout using scTenifoldKnk. (B) Top differentially regulated genes following MYLK network perturbation. (C, D) GO enrichment analyses of network-derived genes. (E, F) KEGG pathway enrichment analyses of network-derived genes. [file Image3.tif]
